# Tumor-associated macrophages display differential protein cargo sorting in extracellular vesicles associated with poor survival in ovarian cancer

**DOI:** 10.1186/s10020-025-01416-x

**Published:** 2026-01-30

**Authors:** Johanna Pörschke, Sophie Heidemann, Hannah P. Nehring, Aina Lluch, Witold Szymański, Florian Finkernagel, Christian Preußer, Aditya M. Bhagwat, Timm J. Stamm, Leah Sommerfeld, Frederik Helmprobst, Rolf Müller, Silke Reinartz, Johannes Graumann, Elke Pogge von Strandmann, María Gómez-Serrano

**Affiliations:** 1 Institute for Tumor Immunology, Center for Tumor Biology and Immunology (ZTI), Philipps-Universität, Marburg, 35043 Germany; 2 Translational Oncology Group, Center for Tumor Biology and Immunology (ZTI), Philipps-Universität, Marburg, 35043 Germany; 3 Institute of Translational Proteomics and Core Facility Translational Proteomics, Biochemical/Pharmacological Center, Philipps-Universität, Marburg, 35043 Germany; 4 Genomics Core Facility, Philipps-Universität, Marburg, 35043 Germany; 5 EV-iTEC Core Facility, Philipps-Universität, Marburg, 35043 Germany; 6 Core Facility for Mouse Pathology and Electron Microscopy - Institute of Neuropathology, Philipps-Universität, Marburg, 35043 Germany; 7 Present address: Department of Immunobiochemistry, Mannheim Institute for Innate Immunoscience (MI3) & European Center for Angioscience (ECAS), Medical Faculty Mannheim, Heidelberg University, Mannheim, Germany; 8 Present address: Department of Urology, Universitätsklinikum Tübingen, Tübingen, 72076 Germany

**Keywords:** Extracellular vesicles, Extracellular particles, Glycosylation, Macrophages, Nano-flow cytometry, Ovarian cancer, Post-translational modification, Tumor-associated macrophages

## Abstract

Ovarian cancer (OC) progression and metastasis are promoted by ascites, which constitutes a central part of the tumor microenvironment (TME). In this fluid, tumor-associated macrophages (TAMs) represent a prominent immune cell type. In addition to tumor and other host cells such as TAMs, ascites is highly enriched in soluble factors as well as extracellular vesicles (EVs). How TAMs contribute to the EV compartment of the OC TME remains, however, underexplored. In this work peripheral blood monocytes from healthy donors were differentiated into monocyte-derived macrophages (MDMs) and polarized into classically activated (M1-like), alternatively activated (M2-like) and TAM-like (by ascites incubation). For all subtypes, serum-free conditioned medium was collected for 24 h and EVs were isolated and characterized by nano-flow cytometry (nFC), label-free mass spectrometry-based proteomics and electron microscopy, among others. Our results demonstrated distinct traits for EV release and cargo across the different macrophage subtypes. Specifically, TAM-like macrophages exhibited impaired release of small EVs and reduced frequency of tetraspanin-positive particles. These EV subpopulations displayed sizing profiles closer to M1-like than to M2-like samples. Also, the low EV release in TAM-like MDMs was accompanied by altered expression of biogenesis-related markers like flotillin-1 (FLOT1) and a decreased N-glycosylation of CD63 protein, which was validated in patient-derived samples. Remarkably, the EV-associated proteome of TAMs displayed significant enrichment in both pro- and anti-inflammatory molecules with clinical value. Markers significantly enriched in the ascites TAM-EV signature were mostly associated with poor prognosis, whereas M1-like EV-related markers (pro-inflammatory) were mostly associated with longer survival. Our results confirmed previous data for proteins like CD163 and MRC1 to be associated to TAM-EVs, while also describing novel candidates with diagnostic (*i.e.,* COLEC12) and/or prognostic (*i.e.,* MSR1) value in plasma. Taken together, our data support a unique secretory profile of TAMs in OC and provide new EV-associated biomarkers with translational impact. Our results pave the way for a better understanding of the mechanisms behind TAM-EV cargo loading and function, and how these cells participate in the TME landscape.

**Supplementary Information:**

The online version contains supplementary material available at 10.1186/s10020-025-01416-x.

## Highlights

Ascites-driven reprogramming of macrophages results in reduced extracellular vesicle (EV) release but enriched pro-tumorigenic cargo.

Tumor-associated macrophages (TAMs) in ovarian cancer (OC) show decreased N-glycosylation of CD63 at the cellular level, further supporting their immunosuppressive profile.

Combination of nano-flow cytometry and proteomics revealed distinct profiles of tetraspanin-subpopulations and differential cargo sorting in macrophage-derived EVs.

High-resolution proteomic analyses of TAM-derived EVs identified a unique signature with potential clinical value in OC.

**Supplementary Information:**

The online version contains supplementary material available at 10.1186/s10020-025-01416-x.

## Introduction

Ovarian cancer (OC) is the most lethal gynecological malignancy (Ledermann et al. 2013), ranking as the fifth deadliest cancer in women worldwide. Within OC, 90% of the cases are of epithelial tumor origin (Kurman et al. 2020). The World Health Organization (WHO) classifies malignant epithelial OC into five major types based on the histopathology, immune profile and molecular analysis: high-grade serous carcinoma (HGSC, 70% of cases), endometrioid carcinoma (10%), clear cell carcinoma (6–10%), low-grade serous carcinoma (5%) and mucinous carcinoma (3–4%) (Kurman et al. 2020; De Leo et al. 2021). This work focuses on HGSC, characterized by early metastasis into the *omentum* together with excessive accumulation of peritoneal fluid within the abdominal cavity, referred to as ascites (Worzfeld et al. 2017). Ascites contains cancer cells (Latifi et al. 2012), immune cells (Reinartz et al. 2016), soluble factors (Kulbe et al. 2012) and extracellular vesicles (EVs) (Peng et al. 2011), which together contribute to tumor invasion, chemoresistance and immune evasion (Ahmed and Stenvers 2013; Kipps et al. 2013). The process of metastasis is supported by both the cellular components of the tumor microenvironment (TME) as well as their released factors, referred to as the tumor secretome (Yang et al. 2025).

EVs are membrane bilayer-encapsulated lipid vesicles released by almost any type of cell into the extracellular space. EVs can be divided into different categories according to their measurable size (small, 50–200 nm; or large, 200–1000 nm), density, cellular origin, molecular markers (*i.e.,* endosomal pathway, membrane budding or apoptosis), function and cargo, which include nucleic acids, proteins and lipids (Doyle and Wang 2019; Kanada et al. 2015). EVs have been demonstrated to play key roles in intercellular communication, yielding functional alterations on recipient cells (Harding et al. 2013). The spectrum of EV function is wide, including tumor progression as well as the stimulation of immune responses through antigen-presentation (Buzas 2023). Recently, the contribution of EVs from host cells to the TME has been described to exceed that of EVs from tumor cells in HGSC (Vyhlidalova Kotrbova et al. 2024), an observation replicating previous findings for the soluble factors of ascites (Sommerfeld et al. 2021; Worzfeld et al. 2018). Among the host cells found in HGSC ascites, tumor-associated macrophages (TAM) are one of the most abundant cell types. Macrophages are mononuclear phagocytic immune cells involved in processes like infection resolution, homeostasis or phagocytosis of foreign substances or cells (Haniffa et al. 2015; Yona and Gordon 2015). Peripheral blood monocytes are thought to be the major source of macrophages (Ginhoux and Jung 2014), recruited to organs and locally differentiated into tissue-specific macrophages. The surrounding environment polarizes these macrophages, which have historically been classified into two major subtypes for their study. On the one hand, classically activated macrophages (M1-like) take part in pro-inflammatory processes by producing cytokines and having pathogen-killing functions, as well as enhanced antigen-presenting capacities (Biswas and Mantovani 2010). Additionally, anti-tumor functions have also been typically attributed to them (Boutilier and Elsawa 2021). On the other hand, alternatively activated macrophages (M2-like) take part in anti-inflammatory processes, parasite infection, tissue remodeling, allergic disease and angiogenesis (Jenkins et al. 2013; Mantovani et al. 2013). Furthermore, they play a relevant immunosuppressive role (Hensler et al. 2020), contributing to tumor growth and metastasis formation. Macrophages are recruited by cytokines produced by tumor cells such as MCP-1 (Monocyte chemoattractant protein 1, also known as CCL2) (Negus et al. 1995), determining their phenotype and activation state (Gordon and Taylor 2005). It has been described that TAMs show a mixed phenotype of M1- and M2-like activation state (Noy and Pollard 2014), which has also been demonstrated in the ascites of OC patients (Reinartz et al. 2016). For instance, high expression levels of the M2-surface marker CD163 (Scavenger receptor cysteine-rich type 1 protein M130) on ascites-derived TAMs associates with an early relapse following first-line therapy of HGSC (Reinartz et al. 2016). Ascites TAMs are mostly thought to derive from peripheral blood monocytes, but tissue-resident macrophages may also be a source of origin (Liu and Cao 2015). With respect to marker expression and activation state, ascites-derived TAMs from OC show similarities to resident peritoneal macrophages (Worzfeld et al. 2017). Both populations express, for instance, high levels of CD163 and CD206 (Macrophage mannose receptor 1, also known as MRC1), as well as genes related to phagocytosis and antigen presentation functions (Worzfeld et al. 2017).

TAMs are important producers of cytokines including interleukins (IL) (*e.g.,* IL6, IL10), CCL18 (C–C motif chemokine 18), CCL22 (C–C motif chemokine 22), TNF-α (Tumor necrosis factor) and TGF-β (Transforming growth factor beta) (Kolomeyevskaya et al. 2015; Rodriguez et al. 2001; Duluc et al. 2009), supporting cancer metastasis, adhesion, invasion and proliferation of OC cells (Worzfeld et al. 2017). In contrast to these well-studied soluble factors, little is known about the contribution of TAM-derived EVs in OC. Macrophage-derived EVs have been described to differ between macrophage subtypes, *e.g.,* through divergent miRNA profiles (Kwon et al. 2020). As TAMs promote an immunosuppressive *milieu* for tumor progression (Wang et al. 2020a), M2-EVs have been mostly used as surrogate models of TAM-EVs. Such EVs have, for example, been involved in T-cell imbalance contributing to an immunosuppressive environment in epithelial OC (Zhou et al. 2018), as well as in the direct promotion of tumor migration and invasion of pancreatic ductal adenocarcinoma (Yin et al. 2019) and gastric cancer (Zheng et al. 2018). Circulating TAM-derived EVs have, however, been reported to show expression profiles associated with M1-like activation state, which controversially hints at antitumor potential (Cianciaruso et al. 2019). Due to these discrepancies and given the limited knowledge about TAM-derived EVs in the context of HGSC (Vyhlidalova Kotrbova et al. 2024), these mediators warrant further analysis. This work thus aimed at the characterization of EVs from different macrophage subtypes, with a focus on ascites-influenced TAMs as key drivers of OC progression. Our results led to improved understanding of EV release and protein cargo in TAMs. Also, we confirmed the association of previous proteins involved in OC progression (Steitz et al. 2020) to macrophage-derived EVs, while depicting new markers with clinical and prognostic value in HGSC.

## Methods

### Ethical considerations

The Center for Transfusion Medicine and Hemotherapy at the University Hospital Gießen and Marburg (UKGM) kindly provided Leukocyte Reduction System (LRS) chambers from healthy blood donors. Ascites was collected from patients with HGSC undergoing surgery at primary diagnosis before receiving any kind of chemotherapy. Acute or chronic infections were exclusion criteria for patient selection. All patients received standard first-line therapy (carboplatin/paclitaxel) subsequently. Maintenance therapy as well as patient characteristics are described in the Supplementary Information (Supplementary Table S1). Collection of samples and analysis were approved by the local ethics committee (No. 205/10) of the UKGM. All donors provided written consent in accordance with the Declaration of Helsinki.

### Human monocyte isolation and differentiation to macrophages

Mononuclear cells from LRS chambers were obtained by Ficoll density gradient centrifugation and CD14 + monocytes were purified by adherence selection. Monocytes were seeded and used for subsequent differentiation in a density of approximately 2 × 10^5^ cells/cm^2^. Cells were differentiated towards M1-like and M2-like monocyte-derived macrophages (MDMs) as previously (Steitz et al. 2020). Briefly, adhered monocytes were cultured 5 days in RPMI1640 (Cat. 61870036, Gibco™, Life Technologies), supplemented with 5% human AB serum (Cat. H4522, Sigma-Aldrich), 1% sodium pyruvate (Cat. S8636, Sigma Aldrich), and 100 ng/mL granulocyte macrophage colony-stimulating factor (GM-CSF, Cat. 300–03, PeproTech®, Thermo Fisher Scientific) for M1-like or 20 ng/mL macrophage colony-stimulating factor (M-CSF, Cat. 574804, BioLegend) for M2-like MDMs. Subsequently, M1-like MDMs were activated by 100 ng/mL lipopolysaccharide (LPS, Cat. L439, Sigma-Aldrich) and 20 ng/mL IFNγ (Cat. 51564.100, Biomol), while M2-like MDMs were stimulated with 20 ng/mL IL-10 (Catalog. 1145, Proteintech) for another 2 days in addition to GM-CSF and M-CSF, respectively. In order to obtain TAM-like MDMs, monocytes were cultured for 7 days in 100% cell-free ascites fluid pool (from 10 HGSC patients), to minimize patient-dependent variability. Patient cohorts used for ascites pool preparation over the course of this study were overall homogenous with respect to histology, staging and age (Supplementary Table S2).

### Flow cytometry analysis

Flow cytometry analysis of MDMs was performed using a FACS Canto II machine with Diva Software (BD Bioscience). Cells were harvested with TrypLE™ (GibcoTM) and subsequently washed with *Staining buffer* (1% fetal bovine serum, FBS, in phosphate-buffered saline, PBS). Polyclonal mouse IgG (1:100, Jackson Immuno Research) was added at least 15 min at 4 °C to block unspecific Fc binding. All MDMs were stained for surface markers using anti-human CD14-FITC (Cat. 130–113–146, Miltenyi), CD86-FITC (Cat. 130–116–159, Miltenyi), CD16-PE-Cy7 (Cat. 25–0168-42, Invitrogen), CD163-PE (Cat. 12–1639-42, Invitrogen), HLA-DR-APC (Cat. 17–9956-42, Invitrogen) and CD206-APC (Cat. 321110, Biolegend) for 30 min at 4 °C, while CCR7-PE (Cat. 560765, BD Bioscience) was incubated at 37 °C. Corresponding isotype controls were derived from BD Bioscience, Miltenyi and Invitrogen, respectively. The gating was performed based on the isotype controls and the percentage of positive cells or the mean fluorescence intensity (MFI) was calculated.

### Cell vitality assays

In order to further evaluate the vitality of macrophage subtypes, the *LIVE-DEAD Cell Imaging Kit* (Cat. R37601, Invitrogen™, Thermo Scientific) was used on adherent MDMs after differentiation/polarization and 24 h starvation, following manufacturer´s instructions. MDMs were washed with PBS once and stained with a volume of 220 μL of staining solution per 24-well. The cells were incubated at room temperature for 5 min and imaged immediately after. The imaging was done as quickly as possible, since the staining eventually starts killing the cells, skewing the data. A total of 7 random fields per donor (*n* = 3) and MDM subtype were taken under Leica DMI3000 B Microscope. Images from green (live) and red (dead cells) channels were separately analyzed by Image J software (*cell counter*).

### Conditioned media collection and EV isolation

Conditioned media were generated from all MDM subtypes. After 7 days of cultivation, polarization media or ascites was removed, cells were washed three times with PBS and serum-free RPMI1640 supplemented with 1% sodium pyruvate was added for 24 h at 37 °C and 5% CO_2_. Time-point zero samples were collected as blank for further analysis. Afterwards, conditioned medium was harvested and cell debris was removed by 300 × g centrifugation for 10 min. Supernatant was then taken and cell organelles were further separated by centrifugation at 2,000 × g for 15 min. Moreover, the supernatant was centrifuged at 10,000 × g for 1 h to remove large particles. All centrifugation steps were performed at 4 °C. Supernatants (10,000 × g) collected from 12-well plates were used for particle release and median size analysis by nano flow-cytometry after one freeze and thaw cycle.

Supernatants from P_100_ dishes were further processed by ultrafiltration (UF) with Amicon filter devices with cut-off of 100 kDa (Cat. UFC910024, Sigma Aldrich) or differential ultracentrifugation (UC) for small EV isolation (herein after indicated only as “EVs” for simplicity). For UF, Amicon filters were washed with PBS once and subsequent loaded with the 10,000 × g supernatants. UF samples were concentrated by 4,000 × g centrifugation for 10 min at 4 °C. The concentrate was diluted with 0.1 µm filtered PBS and concentrated again. These EV-enriched samples were used for subsequent detection of tetraspanin markers (fluorescence labelling).

EV isolation by UC was performed on 10,000 × g supernatants using the Optima XPN-80 ultracentrifuge and SW 32 Ti swinging-bucket rotor (Beckmann Coulter) for 2 h at 110,000 × g and 4 °C. Samples were suspended in filtered PBS (1 mL) and concentrated using the Optima™ MAX-XP ultracentrifuge and TLA-45 fixed-angle rotor (Beckmann Coulter) for 2 h at 110,000 × g and 4 °C. EV pellets were resuspended in 50 µL filtered PBS and stored at −20 °C until further analyses.

### EV characterization

#### Nano-flow cytometry analysis

Nano-flow cytometry (nFC) was used to analyze particle concentration and size of conditioned media (10,000 × g supernatants) as well as concentrated and isolated EVs after UF or UC, respectively. To that purpose, the NanoAnalyzer equipped with a 488 nm laser and two single photon-counting avalanche photodiodes (NanoFCM, Inc.) was used with the calibration settings indicated by the manufacturer and implemented by the EV-iTEC Core Facility Marburg. Briefly, monodisperse silica nanoparticles cocktail (68–155 nm, Cat. S16M-Exo, NanoFCM, Inc.) and 200 nm polystyrene beads (QC Beads, Cat. S08210, NanoFCM, Inc.) with a defined concentration of 2.08 × 10^8^ particles/mL were used for size and concentration calibration, respectively, at a sampling pressure of 1 kPa. This reference allows a single particle-analysis in a range of 40 to 200 nm. Either 0.1 µm filtered PBS or time-point zero samples of respective conditioned media were used for background subtraction. Dilutions of samples were adapted individually to achieve a total number of analyzed particles between 2,500–12,000 events per minute of acquisition (linear range determined by the manufacturer). Samples were analyzed using the NanoFCM software as indicated (NF Profession V1.08 and V2.0, NanoFCM, Inc.).

#### EV staining and fluorescence analyses

Staining was done on EV-enriched samples by UF (3–5 × 10^8^ particles). For comparative analyses, the same number of particles were stained using 200 ng of anti-human CD9-FITC (Cat. 312104, Biolegend), CD63-FITC (Cat. 353006, Biolegend) and CD81-FITC (Cat. 349504, Biolegend). Respective isotype control was checked (Cat. 400110, Biolegend). Mastermixes of the antibodies were prepared in 0.1 µm filtered PBS in advance and centrifuged for 15 min at 10,000 × g at 4 °C prior to application. EVs were stained for 1 h at 37 °C and 600 rpm in the dark. Afterwards, EVs were washed with 0.1 µm filtered PBS (final volume 1 mL) and concentrated by a 110,000 × g centrifugation for 1 h and 40 min at 4 °C using the Optima™ MAX-XP ultracentrifuge and TLA-45 fixed-angle rotor (Beckmann Coulter). PBS was discarded leaving a rest volume of 30 µL to resuspend the EV pellet. Dilutions for optimal particle range acquisition were individually tested to ensure they fit into the linear range of detection determined by the manufacturer (2,500—12,000 events/min), and the percentage of positive events (gated in FITC-A) was calculated. Samples were analyzed using the NanoFCM software (NF Profession V2.0, NanoFCM, Inc.).

#### Detergent lysis control of EVs

Approximately 5 × 10^8^ UC-isolated particles were suspended either in PBS (untreated, UT) or in Triton-X100 solution (final concentration 1% v/v in PBS), vortexed and incubated for 15 min at room temperature (RT). Dilutions for optimal particle range acquisition were based on UT samples (approximately 1:500 dilution, corresponding to 3,000—6,000 events/min). Corresponding Triton solution was acquired in the same dilution and used as background signal. Samples were analyzed using the NanoFCM software (NF Profession V2.0, NanoFCM, Inc.).

#### Transmission electron microscopy

Sample preparation and transmission electron microscopy (EM) analysis were done according to previous work (Thery et al. 2006). Briefly, 10 µL of EV suspension were fixed in an equal amount of 4% paraformaldehyde (PFA) and a small volume was transferred on a Formvar-carbon coated EM grid. Grids were air-dried for 20 min, washed with PBS and incubated for 5 min in 1% glutaraldehyde. Afterwards, the grids were washed eight times with distilled water (2 min) and transferred to a uranyl-acetate solution, followed by a 10 min incubation on ice in a 4% uranyl acetate and 2% methyl cellulose mixture in a 1/9 ratio, respectively. Excess fluid was removed and the grids were air-dried for 5–10 min. EVs were imaged with a Zeiss EM 900 at 80 kV, fitted with a 2 k slow-scan CCD camera (TRS). EV size estimation was done with ImageJ software evaluating n ≥ 150 single events (per sample type).

### Protein analyses

#### Cell lysates and protein quantification

For protein isolation, MDMs seeded in 12-well plates or P_100_ dishes were used after conditioned media collection. Ex vivo patient-derived TAMs were isolated as in previous studies (Reinartz et al. 2016; Worzfeld et al. 2018). Macrophages were washed with PBS and suspended in radioimmunoprecipitation assay buffer (RIPA) supplemented with protease inhibitor (Cat. P8340, Sigma Aldrich) and phosphatase inhibitor mix (sodium pyrophosphate decahydrate, 20 mM; b-Glycerol phosphate, 50 mM; sodium orthovanadate, 50 mM; sodium Fluoride, 100 mM). Lysis was done upon scrapping and vortexing, followed by mixing and incubating the sample on ice for 15–20 min. Afterwards, the cell lysate was centrifuged at 17,000 × g for 10 min at 4 °C and the protein supernatant was collected. Protein concentration was estimated by the bicinchoninic acid assay (BCA, Pierce™, Thermo Scientific) and stored at −20 °C until further analyses.

#### Immunoblotting

Cell lysates (5–15 μg protein) were homogenized in RIPA as indicated before. EV samples were homogenized in RIPA (10X) solution and incubated on ice for 15 min, followed by a freeze and thaw cycle at −20 °C. Prior to loading, *Laemmli buffer* (5X, 255 mM Tris–HCl, 50% glycerin, 5% (w/v) sodium dodecyl sulfate – SDS –, 0.05% (w/v) bromophenol blue) was added and samples were boiled for 5 min at 95 °C. *Laemmli buffer* was additionally supplemented with 250 mM dithiothreitol (DTT) when reducing conditions for protein detection were necessary. All samples were resolved by SDS-PAGE (sodium dodecyl sulfate polyacrylamide gel electrophoresis) on 10% gels and transferred to Hybond ECL nitrocellulose membranes according to standard procedures. Total protein staining (*MemCode™ 520 Reversible Protein Stain Kit*, Cat. 24580, Thermo Fisher Scientific) was performed in order to check proper loading and membranes were blocked for 1 h with 2.5% (w/v) non-fat milk powder or 5% bovine serum albumin (BSA, Cat. 8076.4, Carl Roth) in Tris-buffered saline (TBS) buffer containing 0.2% Tween-20 (TBS-T). Primary antibody solutions were probed over-night (ON) at 4 °C (Supplementary Table S3). Blots were washed and incubated with either anti-mouse IgG-HRP-conjugated secondary antibody (Cat. 7076S, Cell Signalling, Leiden, Netherlands) or fluorescently labeled anti-rabbit IgG-IRDye 800CW (Cat. 926–32211, LI-COR Biosciences) or anti-mouse IgG-IRDye 680RD (Cat. 926–68070, LI-COR Biosciences) at 1:10000 dilution in TBS-T for 1 h. Both detection methods were combined. Chemiluminescent bands were detected by incubation with *Immobilon Forte Western HRP substrate Millipore* (Cat. WBLUF0500, Merck, Germany). Prior to fluorescence detection, membranes were washed with TBS solution (without detergent) to prevent background interferences. Both methods were visualized at different acquisition times using a ChemiDoc MP system (Bio-Rad Laboratories, Inc.). Optical densities of the immunoreactive bands were measured using *Image Lab* analysis software version 5 (Bio-Rad Laboratories, Inc.).

#### Digestion with N-glycosidase F

MDM lysates were collected as described above and treated with the *PNGase F Glycan Cleavage Kit* (Cat. A39245, Gibco, Thermo Scientific) following manufacturer´s recommendations. Briefly, 25 μg of total protein was brought to a reaction volume up to 35.5 μL with HPLC-grade water and incubated at 50 °C for 1 h with PNGase F master mix (*c.a.,* 150 U/μg protein) and then centrifuged briefly. Samples without PNGase F enzyme were used as negative control and equally loaded for immunoblotting analyses.

#### EV sample preparation and digestion for proteomics

MDM-derived EVs were isolated by UC as described above. Due to the marked differences in yield between MDM subtypes, the input for protein digestion was normalized to 2 × 10^9^ particles/donor. M1-like, M2-like and TAM-like EV samples were analyzed in unpaired biological replicates (*n* = 3 per group).

Samples were lysed by incubation with sodium lauroyl sarcosinate (SLS) solution at a final concentration of 4% at 95 °C for 10 min, followed by reduction and alkylation through addition of DTT to a final concentration of 10 mM with incubation at 95 °C for 10 min, and iodoacetamide (IAA) to a final concentration of 13 mM with incubation for 30 min at 25 °C, respectively. Peptidolysis was achieved using a modified version of the SP3 method (Hughes et al. 2019) as previously implemented for EV preparations (Pedro et al. 2023). Briefly, protein binding was performed in a final concentration of 70% anhydrous acetonitrile (ACN) solution at neutral pH with subsequent washes with 70% ethanol and 100% ACN. After ACN removal, beads were resuspended in 50 μL of 50 mM triethylammonium bicarbonate (TEAB) buffer and 0.5 ug of trypsin (Promega, Madison, Wisconsin, USA) was added. Protein digestion was performed ON, at 37 °C. Next, sample volumes were reduced to approximately 5 μL in a SpeedVac concentrator (Eppendorf). Peptide binding to beads was initialized by the addition of 100% ACN to a final concentration exceeding 98%. Beads were washed twice using the same solvent. Peptides were eluted by the addition of 40 μL of 0.1% formic acid and transferred to analytical vials. Peptide concentration was estimated using the fluorimetric Pierce™ Quantitative Peptide Assay and volumes were adjusted to achieve equal concentrations.

#### Mass spectrometric data acquisition

Peptides were analyzed by liquid chromatography/tandem mass spectrometry (MS) carried out on a Exploris 480 instrument connected to an Ultimate 3000 rapid separation liquid chromatography (RSLC) nano instrument and a nanospray flex ion source (all Thermo Fisher Scientific, Waltham, USA). Peptide separation was performed on a reverse-phase high-performance liquid chromatography (HPLC) column (75 μm by 42 cm) packed in-house with C18 resin (2.4 μm; Dr. Maisch HPLC GmbH, Ammerbuch, Germany). The peptides (approximately 300 ng) were first loaded onto a C18 precolumn (preconcentration set-up) and then eluted in the backflush mode with a gradient from 98% solvent A (0.15% formic acid, FA) and 2% solvent B (99.85% ACN and 0.15% FA) to 25% solvent B over 65 min, continued from 25 to 35% of solvent B for another 24 min. The flow rate was set to 300 nL/min. The data were acquired in a data-independent mode (DIA) for the initial label-free quantification, and study was set to obtain one high-resolution MS scan at a resolution of 120,000 (m/z 200) with the scanning range from 350 to 1400 m/z followed by DIA scans with 45 fixed DIA windows with the width of 14 m/z (1 m/z overlap from neighboring windows), ranging from 320 to 950 m/z at a resolution of 15,000. The automatic gain control was set to 300% for MS survey scans and 3000% for DIA scans. Detailed settings for the used method can be found in the “DIA-90 min-49w.meth” uploaded along with the mass spectrometric raw data to the ProteomeXchange Consortium with dataset identifier: PXD065421, via the MassIVE partner repository (https://massive.ucsd.edu/, MassIVE ID: MSV000098313; doi:10.25345/C50V89W2H).

#### Spectra identification

Peptide spectrum matching and label-free quantitation were performed using DIA-NN (Demichev et al. 2020) using library-free search against the *Homo sapiens* Uniprot database (20,407 Swiss-Prot entries, April 2023), parametrized as documented in the MassIVE repository. In brief, output was filtered at 0.01 false discovery rate (FDR) on the precursor level. Deep learning was used to generate an in silico spectral library for library-free search. Fragment m/z was set to a minimum of 200 and a maximum of 1,800. In silico peptide generation allowed for N-terminal methionine excision, tryptic cleavage following K*, R*, a maximum of one missed cleavage, as well as a peptide length requirement of seven amino acids minimum and a maximum of 30. Cysteine carbamidomethylation was included as a fixed modification and methionine oxidation (maximum of two) as a variable modification. Precursor masses from 300 to 1,800 m/z and charge states one to four were considered. DIA-NN was instructed to optimize mass accuracy separately for each acquisition analyzed and protein sample matrices were filtered using a run-specific protein q-value.

#### Downstream proteomic data processing

Data analysis and statistics were carried out on DIA-NN’s “report.tsv” using the R package *Autonomics* (Version autonomics_1.15.143) (Bhagwat et al. 2025), including proteins with a protein q-value < 0.01 and requiring detection of three or more precursors (Np ≥ 3) in at least half of the donors as additional filtering criteria. Median centered raw intensities MaxLFQ (Cox et al. 2014) were used for quantification and missing values imputed. The imputed protein intensities mimic a lower value of detection of the mass spectrometer during the measurement and are derived from the distribution of the values measured. Differential expression analysis was evaluated by *Autonomics*, employing Bayesian moderated t-testing as implemented by limma (Ritchie et al. 2015). Proteins with differential abundance were determined considering a cut-off of FDR-adjusted *p* value < 0.05.

### Bioinformatics and statistical analyses

#### Protein list visualization and functional enrichment analyses

Venn diagrams were analyzed by BioVenn software (https://www.biovenn.nl/index.php) (Hulsen et al. 2008). TAM-EV signature proteins (*n* = 225) were examined by over-representation analysis (ORA) using the *enrichGO* function from the *clusterProfiler* package (version 4.16.0) in order to identify enriched *Gene Ontology* (GO) terms across *Biological Process* (BP), *Cellular Component* (CC), and *Molecular Function* (MF) categories. The background set consisted of all encoding genes for the proteins detected in the merged dataset (*n* = 2,870 proteins). ORA parameters included *p-value cut-off* = 0.05, *q-value cut-off* = 0.20, Benjamini–Hochberg (BH) correction, minGSSize = 15, and maxGSSize = 500. The analyses were performed using R software (version 4.5.1) within RStudio (version 2024.12.1 + 563).

#### MISEV categorization and protein terms

In order to get an overview of the composition of the EV-associated proteome, a list of gene-encoded proteins was annotated and categorized according to the recent MISEV (Minimal Information for Studies of Extracellular Vesicles) 2023 guidelines (Welsh et al. 2024). Our list (*n* = 1952 hits) extends and updates the previous one published by Vyhlidalova Kotrbova et al. (Vyhlidalova Kotrbova et al. 2024) (Supplementary Table S4). MISEV category 1 (transmembrane proteins associated with endosomal or plasma membranes) and category 2a (cytosolic proteins linked to EV biogenesis), were considered as representative markers for a canonical “EV panel” (*n* = 129 hits). Due to the nature of their classification, category 3 and category 5 had originally overlapping terms (Welsh et al. 2024). Thus, we decided to stick in category 3a only for “lipoproteins” and subclassified the rest of secreted proteins in category 5 (secreted and/or associated with the EV-corona). In order to extend the potential list of non-EV co-isolated structures (NVEPs) in category 3, a list of exomere/supermere-enriched components was prepared based on recent publications (Tosar et al. 2022; Zhang et al. 2018, 2021) (category 3c, *n* = 185 hits). Finally, as “mitoEVs” have been recently described to have a significant representation in the EV landscape (Zhou et al. 2023a), hence the most recent version of MitoCarta database (Rath et al. 2021) was incorporated as category 4b. UniProt accession codes for gene-encoded proteins were retrieved from *UniProtKB Reviewed database* (latest version 2025–04–10, https://www.uniprot.org/).

#### Statistical tests

Differential proteomics studies were evaluated for significance, as indicated in the respective section. GraphPad Prism (version 10.5.0) was used to perform statistical analyses, which are further detailed in the figure captions. Shapiro–Wilk test was used to check the normality distribution of the data. Normally distributed data were analysed using paired/unpaired t-tests for two-group comparisons or one-way/two-way ANOVA with Tukey's post hoc test for multiple-group comparisons. When normality could not be tested or for non-normally distributed data, Mann–Whitney tests were used for two-group comparisons, while Friedman/Kruskal–Wallis tests with Dunn's post hoc test were used for multiple-group comparisons including paired or unpaired samples, respectively.

## Results

### Ascites-driven reprogramming of monocytes into tumor-associated macrophages (TAMs) yields reduced particle release

**Fig. 1:**
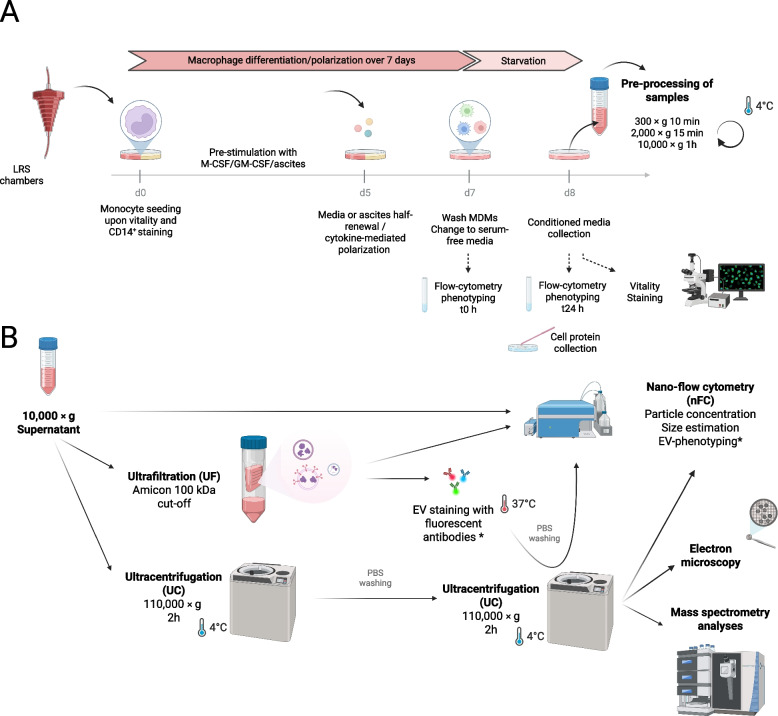
Study design. **A** Sample collection. Monocytes were isolated from PBMCs obtained from peripheral blood from healthy donors collected in Leukocyte Reduction System (LRS) chambers. Cells were differentiated to monocyte-derived macrophages (MDMs) as described in the Material & Methods section. After seven days, polarized MDMs (M1-, M2- and TAM-like) were washed and incubated in serum-free media for additional 24 h. Cells were characterized by flow-cytometry at time points 0 h and 24 h (before and after conditioned medium collection). Additionally, cell vitality was evaluated at the 24 h time point and MDMs were harvested for analysis of the cellular protein lysates. Conditioned media were pre-processed by serial centrifugation prior to EV isolation. **B** Analyses and processing of small EV-enriched samples. Estimation of particle concentration and size were carried out by nano-flow cytometry (nFC) in serum-free conditioned media (10,000 × g supernatants), as well as in EV-enriched samples obtained by ultracentrifugation (UF) and ultracentrifugation (UC), both. UF-enriched samples were stained with fluorescently labelled antibodies, washed, ultracentrifuged and analyzed by nFC (*). UC-isolated samples (unstained) were further analyzed by nFC, electron microscopy and mass spectrometry-based proteomics. Figure created in BioRender: https://BioRender.com/3rvk5ln

We designed a detailed experiment to collect EVs released by M1-, M2- and TAM-like MDMs (Fig. 1). After differentiating monocytes over 7 days using well-established protocols (Steitz et al. 2020), conditioned media from macrophages were collected under serum-deprived conditions (Fig. 1A), preventing FBS-derived contamination (Lehrich et al. 2021). To validate our experimental approach not only prior to but also after EV collection, we checked cellular morphology and measured the expression of M1- (CD86, CCR7, HLA-DR) and M2-markers (CD16, CD163, CD206) in the corresponding subtypes (Supplementary Figure S1). Monitoring of cell morphology excluded major starvation effects on MDMs in the course of EV collection (Supplementary Figure S1A). M1- and M2-markers were expressed as expected in the corresponding polarized M1- and M2-like MDMs, and TAM-like MDMs presented with a M2-phenotype with high levels of CD16, CD163 and CD206 markers (Supplementary Figure S1B). Further, flow-cytometry analyses indicated that M1-like macrophages did not suffer significant changes with respect to any of the surface markers tested in the 24 h course of starvation. In the case of M2-like MDMs, a significant decrease of CD16 and CD163 was observed after serum-deprivation, while the M2c phenotype appeared maintained (Supplementary Figure S1B). TAM-like macrophages in particular, did not show any significant decrease in any of the markers tested, but an increase in HLA-DR and CD206 markers (Supplementary Figure S1B). Based on these observations, we judged the experimental setup sufficiently robust to proceed with the subsequent analyses. As recommended by the MISEV guidelines (Welsh et al. 2024), a set of orthogonal methods for isolating and characterizing EVs was combined (Fig. 1B).

**Fig. 2:**
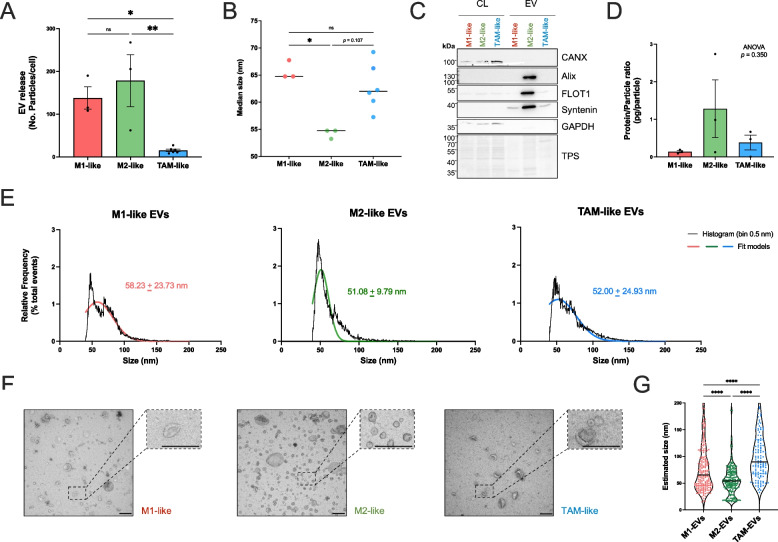
Particle release and size differ between macrophage subtypes. Conditioned media (serum-free) of different macrophage subtypes were collected after 24 h. Cell debris and large particles were removed and small EVs were isolated using two sequential ultracentrifugation (UC) (110,000 × g, 2 h) steps. **A** Particle concentration was estimated by nano-flow cytometry (nFC) (NanoFCM Software V2.0) and EV release was normalized to the number of monocytes seeded. Bar plots represent the average ± SEM (*n* = 3–6 donors). **B** Median size (nm) of particles as determined by nFC. Dots represent individual donors (*n* = 3–6) and lines indicate the median values. **C** Immunoblot of MDM-derived EV samples isolated by UC using the antibodies indicated. Uncropped images are available in Supplementary Figure S2. **D** Estimated protein/particle ratio (pg/particle) in MDM-derived EV samples. Average ± SEM (*n* = 3 donors). **E** Size distribution of particles represented in panel B (bin size 0.5 nm, black line). Non-linear gaussian fit curves are plotted for visualization (M1-, red; M2, green; TAM-like, blue). Calculated means ± SD are indicated. **F** Electron microscopy (negative staining) of MDM-derived EVs isolated by UC. Representative pictures are shown. **G** Violin plots displaying the size estimation of EVs detected by electron microscopy. Measurements were done with ImageJ software. Scale bar, 250 nm. Statistical significance was tested by ordinary one-way ANOVA (followed by Tukey *post-hoc* test) (**A**, **D**) and Kruskal–Wallis test (followed by Dunn *post-hoc* test) (**B**, **G**). ns, non-significant; *, *p* value < 0.05; **, *p* value < 0.01, ****,* p* value < 0.0001. CANX, calnexin; CL, cell lysates; EV, extracellular vesicles; FLOT1, flotillin-1; GAPDH, glyceraldehyde-3-phosphate dehydrogenase; TPS, total protein staining

UC-isolated samples confirmed the presence of small EVs from all MDMs (Fig. 2). Strikingly, our data indicated TAM-like cells to release a very low number of particles (approx. 15 particles/cell), while M1-like and M2-like macrophages released similar levels with an average estimate of 138 and 178 particles/cell, respectively (Fig. 2A). Significant differences in the median size of these particles were detected (Fig. 2B), indicating a smaller median size of M2-like derived samples (approx. 55 nm) in comparison to both M1-like or TAM-like (approx. 65 and 62 nm, respectively) samples. Additionally, we evaluated the enrichment of EV-markers like Alix (Programmed cell death 6-interacting protein), FLOT1 (Flotillin-1) and Syntenin; the presence of cytosolic markers like GAPDH (Glyceraldehyde-3-phosphate dehydrogenase), and excluded the detection of non-EV markers (*i.e.,* Calnexin) in UC preparations by immunoblotting (Fig. 2C, Supplementary Figure S2). Significant decrease in particle detection upon triton treatment (fold-change −0.76 ± 0.07, *p* < 0.0001) further confirmed the presence of EVs in our samples (Supplementary Figure S3). It is noteworthy that M2-like samples showed higher levels of EV markers in comparison to M1- or TAM-like derived particles in protein normalized gels. Although not reaching significance, a tendency of increased protein/particle ratio in M2-EVs may partially explain these observations (Fig. 2D). Results from nFC showed that both size distributions of M1-like and TAM-like derived EVs (50 to 200 nm) were wider and similar (Fig. 2E), as in comparison to M2-like EVs, which showed a higher frequency of smaller particles. EM analyses also supported these observations (Fig. 2F), confirming the smaller size of M2-like EVs and a higher frequency of larger vesicles in TAM-like preparations (Fig. 2G). Furthermore, the smaller size of M2-like EVs as well as the low EV release by TAMs in comparison to both M1/M2 samples were also recapitulated when evaluating particle concentration directly in conditioned media (Supplementary Figure S4), as well as in EV-enriched samples prepared by UF (Supplementary Figure S5).

As the low EV release observed in TAM-like cells was unexpected, we checked whether these remarkable differences were related to alterations in the vitality of the cells following for 24 h (Supplementary Figure S6). *LIVE/DEAD* staining of MDMs after EV collection (Supplementary Figure S6A) revealed that the relative proportion of vital macrophages to remain above 95% in all subtypes, though with significant reduction in TAM-like *vs*. M2-like cells (97.67 ± 1.20% *vs*. 99.78 ± 0.11%) (Supplementary Figure S6B). Neither this, nor the limited differences in macrophage density (Supplementary Figure S6C), could explain, however, the approximate eightfold decrease in particle release per cell in TAM-like *vs*. M2-like macrophages observed (Fig. 2A).

Altogether, our results showed that EV release in macrophages is highly influenced by the surrounding environment. Under our experimental conditions, pro-inflammatory (M1-like) or anti-inflammatory (M2-like) pre-conditioning did not yield significant differences in the number of EVs released by macrophages, but subtype-specific sizing profiles. With particular relevance to the TME, ascites-reprogramming was shown to affect EV release in macrophages, potentially hinting at a significant modulation of EV biogenesis in TAMs.

### Macrophage subtypes differ in EV-subpopulations, reflecting compromised release of small EVs in TAMs

**Fig. 3:**
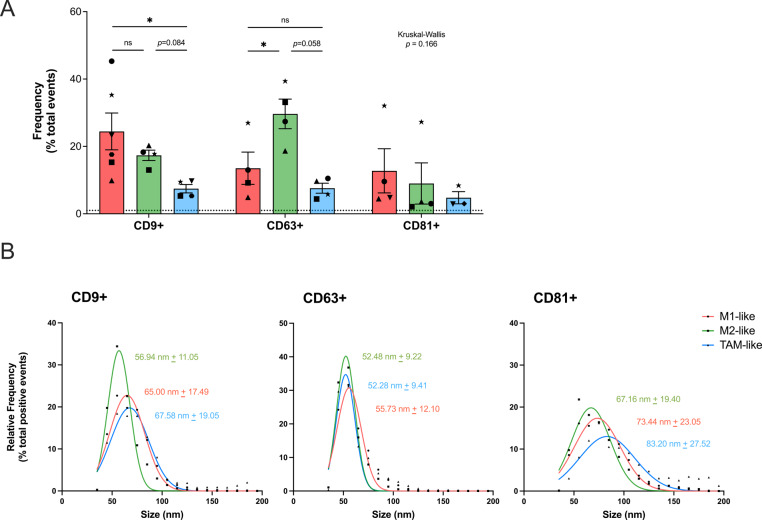
Phenotypic analyses of macrophage-derived EVs. The phenotype of M1- (red), M2- (green) and TAM-like EVs (blue) was analyzed using nano-flow cytometry (nFC) immunofluorescence analyses. **A** Staining results for CD9 +, CD63 + and CD81 + EV subpopulations released into serum-free RPMI media (*n* = 4–6 donors, indicated by different symbols). Equal number of particles per donor and marker were used as staining input to prevent technical biases. Note that due to the low number of TAM-like EVs, not all samples are paired. Statistical significance was tested by Kruskal–Wallis (including Dunn´s *post-hoc* test) for CD9 and CD81 data, and RM one-way ANOVA using Geisser-Greenhouse correction (followed by Tukey´s *post-hoc* test) for CD63 data. *, *p* value < 0.05; **, *p* value < 0.01. **B** Size profile of CD9 +, CD63 + and CD81 + events (within 40–200 nm range). Data represent the relative frequency of total gated, positive events with a bin size of 10 nm (corresponding values from panel **A**). Non-linear gaussian fit curves are plotted for visualization of EV size profiles between macrophage subtypes. Estimated averaged mean values ± SD are indicated in red, green and blue for M1-, M2- and TAM-like positive events, respectively

**Fig. 4:**
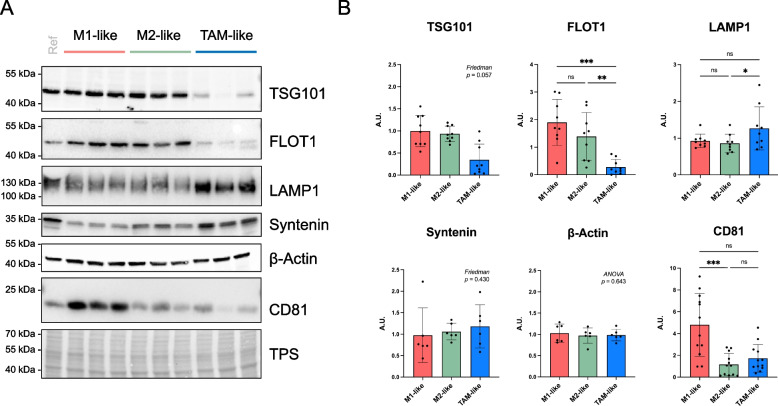
Analysis of EV-related proteins in macrophages. Selected candidates were studied in MDM cellular lysates by SDS-PAGE combining reducing and non-reducing, denaturing conditions (for further details see Supplementary Table S3). **A** Representative images of immunoblots. Three paired samples are shown (identical monocyte donor origin). **B** Relative protein abundance levels detected in M1- (red), M2- (green) and TAM-like (blue bars) samples (*n* = 6–9 donors). Bars represent mean ± standard deviation (SD). Values were normalized to a reference sample and statistical significance was tested by Friedman test including Dunn´s multiple comparisons (for TSG101, Syntenin, CD81 data) or RM one-way ANOVA using Geisser-Greenhouse correction (followed by Tukey´s *post-hoc* test) (for FLOT1, LAMP1, β-Actin data). *, *p* value < 0.05; **, *p* value < 0.01; ***, *p* value < 0.001. A.U., arbitrary units; FLOT1, flotillin-1; LAMP1, lysosome-associated membrane glycoprotein 1, TPS, total protein staining; TSG101, tumor susceptibility gene 101 protein, ESCRT-I complex subunit; Ref, reference sample consisting on non-polarized, Mⱷ, MDMs. Uncropped images are available in Supplementary Figure S8

**Fig. 5:**
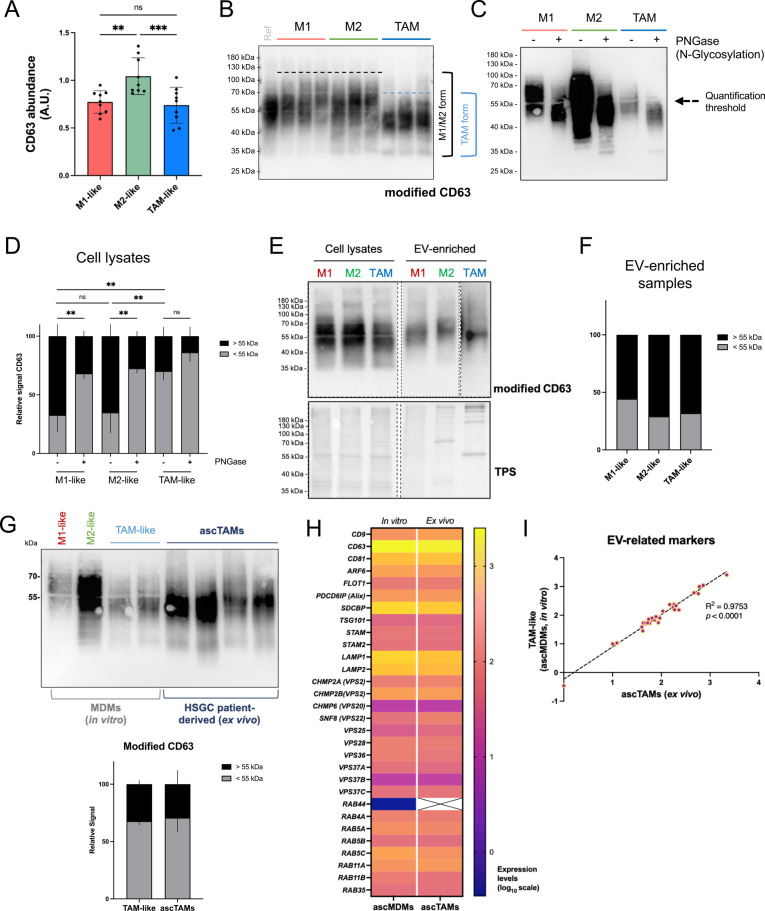
Analysis of CD63 protein in macrophages and derived EVs. CD63 was studied in cellular and EV lysates by SDS-PAGE under non-reducing, denaturing conditions, followed by immunoblotting (see Supplementary Table S3 for further details). **A** Relative CD63 modified protein abundance levels detected in M1- (green), M2- (red) and TAM-like (blue bars) samples (*n* = 9 donors). Bars represent mean ± standard deviation (SD). Values were normalized to a reference sample and statistical significance was tested by RM one-way ANOVA using Geisser-Greenhouse correction (followed by Tukey´s *post-hoc* test). **B** Representative image of CD63 protein detection in the different MDM subtypes. Three paired samples are shown (identical monocyte donor origin). **C** Detection of CD63 in un-treated (-) or PNGase-treated (+) MDM whole cell protein lysates from a representative donor. The downshift in the smear represents loss of N-glycosylation. **D** Relative quantification of CD63 signal modified by PNGase treatment (*n* = 3 donors) in MDM lysates. In order to visualize the shift in the post-translational modification for N-glycosylation, the signal above (black) or below (grey) 55 kDa was quantified and plotted against the whole signal for each lane (mean ± standard deviation, SD). Statistical significance was tested by two-way ANOVA (followed by Tukey´s *post-hoc* test). **E** Detection of CD63 in macrophage-derived EV samples. Results show the detection of CD63 in cell lysates (CL) and EV-enriched samples by UF (pool of *n* = 3 donors) (EV). **F** Relative quantification of the CD63 signal in EV-enriched samples. **G** *Upper panel*: immunoblot detection of CD63 protein in cell lysates from in vitro monocyte-derived macrophages (TAM-like MDMs) *vs*. ex vivo ascTAMs obtained from high-grade serous carcinoma (HGSC) patients (*n* = 4). *Lower panel*: relative quantification of the N-glycosylation pattern in TAM lysates. **H** Heat-map representing the expression levels (RNA-seq, log_10_ scale) of EV-related markers RNAseq analyses partially published in *Sommerfeld, *et al*.* (2021, 2022). **I** Correlation analysis of log_10_-transformed expression levels of the selected EV markers between in vitro TAM-like MDMs (ascMDMs) and ex vivo (ascites-derived TAMs, ascTAMs). Statistical significance was calculated by simple linear regression. Cropped images corresponding to different exposition times are shown due to the differences in the relative abundance (uncropped and different exposition images are shown in Supplementary Figure S9). A.U., arbitrary units; TPS, total protein staining; Ref, reference sample based on non-polarized, Mⱷ, MDMs. *, *p* value < 0.05; **, *p* value < 0.01; ***, *p* value < 0.001

We next evaluated the relative frequency of tetraspanin (CD9, CD63, CD81) positive subpopulations at the single particle level by nFC (Fig. 3, Supplementary Figure S7). Our results showed that the relative frequency of CD9 + EVs was higher in M1-like preparations as compared to TAMs, CD63 + EVs were most prevalent in M2-like samples, and CD81 + showed no significant differences between subtypes (Fig. 3A). Remarkably, TAM-like macrophages showed a very low number of tetraspanin-positive events (< 10% on average for all markers) (Fig. 3A, Supplementary Figure S7). When dissecting the size distribution of the different EV-subpopulations, CD9 + and CD81 + events showed divergent sizing patterns between MDM subtypes (Fig. 3B), whereas CD63 + events showed a clear overlap between the curves (mean size of around 52–56 nm in all subtypes). In both CD9 + and CD81 + subpopulations, M1- and TAM-like macrophages displayed larger vesicles than M2-like cells. Also, CD81 + EVs presented a larger mean size in comparison to CD9 + or CD63 + EVs in all subtypes (Fig. 3B). When checking for the expression of other biogenesis-related factors, we detected alterations at the cellular level of TAM-like macrophages (Fig. 4A, Supplementary Figure S8). For instance, TSG101 (Tumor susceptibility gene 101 protein, ESCRT-I complex subunit) showed a tendency of downregulation in TAMs (Friedman *p* value = 0.057), and FLOT1 (lipid-binding endosomal protein) was significantly reduced in comparison to both M1- or M2-like macrophages (Fig. 4B). In contrast, LAMP1 (Lysosome-associated membrane glycoprotein 1) was significantly more abundant in TAMs in comparison to M2-like macrophages, but not different between M1- and M2-like MDMs. Syntenin (a late endosome protein) and β-Actin (cytoskeleton component) showed comparable levels in all macrophages studied (Fig. 4B). Tetraspanin abundance at the cellular level was also assessed, showing significantly higher CD81 levels in M1-like macrophages compared to M2- or TAM-like cells (Fig. 4B), mimicking to some extent the relative frequency of CD81 + EVs (Fig. 3A). Similarly, CD63 was significantly more abundant in M2-like macrophages in comparison to M1- and TAM-like cells, which showed comparable levels (Fig. 5A). This pattern also resembled nFC results, where M2-like EVs exhibited the highest frequency of CD63 + events (Fig. 3A). Altogether, TAM-like EVs showed the lowest abundance in all tetraspanin-positive EV-subpopulations, concomitant with a general low particle release. Additionally, protein expression of specific biogenesis-related markers like TSG101, FLOT1 or LAMP1 further supported the EV release of TAM-like cells to differ from the other MDMs. Moreover, sizing profiles of CD9 + and CD81 + subpopulations pointed to larger similarity between M1- and TAM-like EVs, while M2-like vesicles indicated a smaller profile with higher frequency of CD63 + EVs.

### TAMs show decreased N-glycosylation of cellular CD63 while favoring EV loading of increased N-glycosylated form

CD63 in TAM-like macrophages showed a slightly shifted, smeary pattern, suggesting altered post-translational modification (PTM) in comparison to M1- or M2-like MDMs (Fig. 5B). PNGase treatment confirmed this shift was due to reduced N-glycosylation of the native form of CD63 in TAMs (Fig. 5C, D). Additionally, CD63 was less abundant in TAM-like EVs in comparison to M1- or M2-like EVs (Fig. 5E, Supplementary Figure S9). Notably, the higher N-glycosylated form was enriched in all EV samples (Fig. 5E, F). These findings suggest that the highly N-glycosylated form of CD63 may be preferentially sorted into macrophage-derived EVs. Remarkably, primary ascites-derived patient TAMs (ascTAMs) showed a CD63 N-glycosylated status similar to ex vivo differentiated TAM-like cells (ascMDMs) (Fig. 5G). Furthermore, we investigated to what extend the in vitro system for TAM-like MDMs reflects the phenotype of primary ascites-derived TAMs (ascTAMs) with respect to EV biology. To this end, we checked the transcript level of 30 selected markers related to EV biogenesis markers including tetraspanins (*i.e., CD9, CD63, CD81*), members of the ESCRT complex (*i.e., VPS25, VPS38, CHAMP2A, TSG101*), as well as intracellular membrane trafficking proteins like Rab GTPases (*i.e., RAB4A, RAB5, RAB11*), in datasets we published previously (Sommerfeld et al. 2021, 2022) (Fig. 5H). The transcript level of these markers in ex vivo ascTAMs significantly correlated with in vitro TAM-like cells (ascMDMs) (*p* < 0.0001) (Fig. 5I). The likelihood of observing a correlation this good between random gene sets of the same size in this comparison was *p* = 0.003 (35 out of 100.000 bootstrapped simulations), supporting preserved gene expression of EV-related markers between ascTAMs and TAM-like cells.

Thus, our data confirm a reduced cellular N-glycosylated status of CD63 on TAMs in comparison to classically (M1) and alternatively activated (M2) macrophages, both in patients and in our in vitro model, providing additional evidence for ascMDMs as a suitable system for studying TAM-EV contribution into the TME.

### The EV-associated proteome reflects differential cargo routes in macrophages

**Fig. 6:**
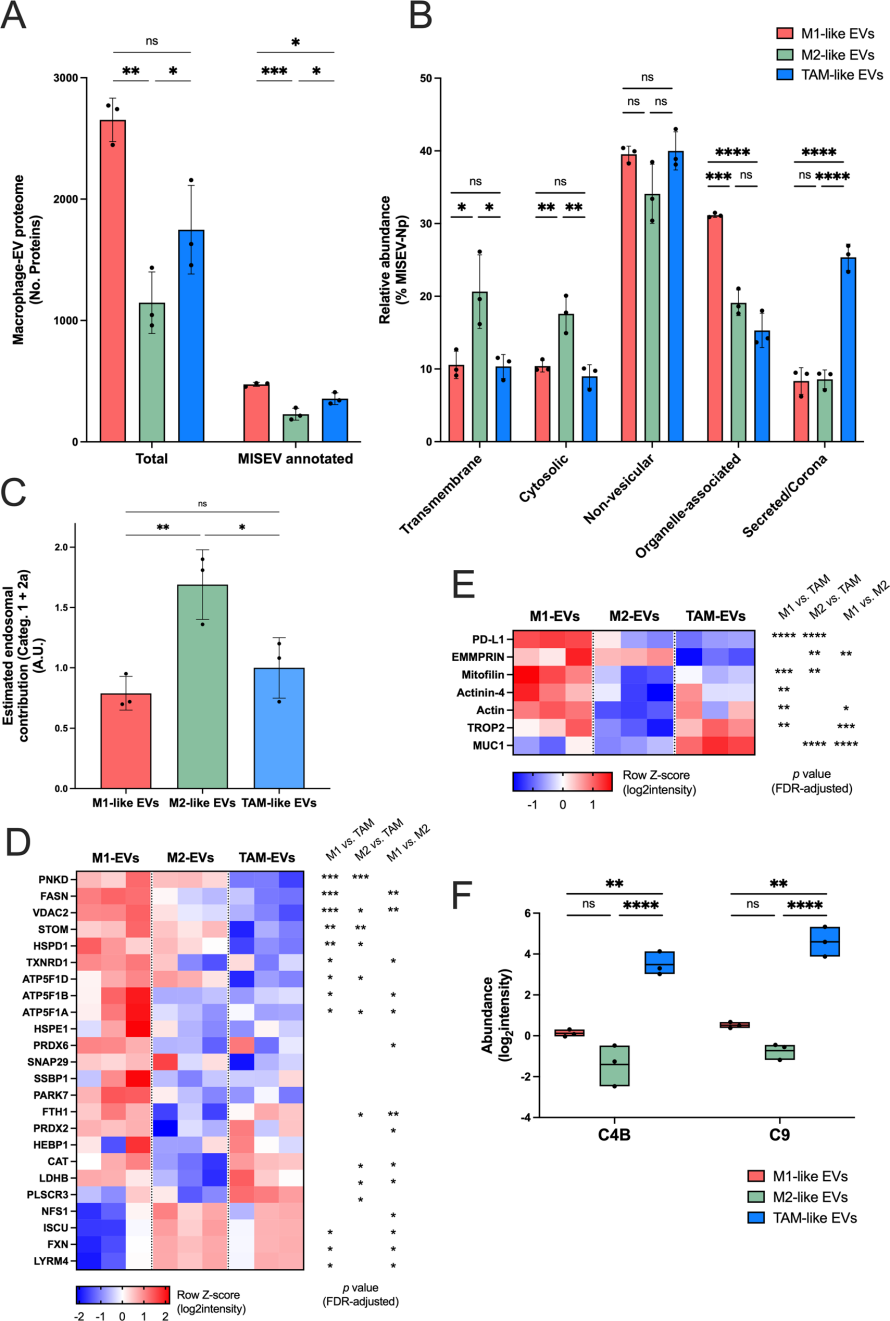
Proteomic characterization of macrophage-derived EVs. **A** Number of protein groups identified across EVs derived from M1-like (red), M2-like (green) and TAM-like (blue) macrophages (*n* = 3 donors per group). Total protein list is depicted in Supplementary Table S5. MISEV-annotated terms are summarized in Supplementary Table S7. **B** Graph bars showing the relative contribution of main categories based on the % of number of precursors (Np) of MISEV annotated proteins. Subcategory evaluation is depicted in Supplementary Figure S11. **C** Estimation of the small EV contribution (endosomal origin) in EV preparations from macrophages. A ratio was calculated based on the relative abundance of proteins belonging to categories 1 and 2a (endosomal origin) (%Np) normalized by the average of TAM-like samples. Bars indicate mean ± SD. Averaged values were analysed and statistically tested by ordinary one-way ANOVA (followed by Tukey´s post-hoc test). *, p value < 0.05; **, p value < 0.01; ***, p value < 0.001; ****, p value < 0.0001. **D** Mitochondrial component of macrophage-derived EVs. Heatmaps showing the differential abundance of mitoproteins across samples. Only hits of the macrophage-EV core are shown. **E** Relative abundance of large EV markers. Color scale indicates the row Z-score (log2-normalized intensity). **F** Representative secreted corona proteins (category 5). Box-plots indicating the relative abundance (log2-normalized intensity) of C4B (complement C4-B) and C9 (complement component 9) across macrophage-derived EV samples. Statistical significance was evaluated between groups by limma t-test (FDR-adjusted p value). *, p < 0.05; **, p < 0.01; ***, p < 0.001; ****, p < 0.0001

The results presented above suggested that EV biogenesis differs between macrophage subtypes, with an increase of smaller EVs in M2-like macrophages and a generally decreased release in TAMs. These differences were already reflected in the differential cytometry profiles of tetraspanin-positive subpopulations, however, whether this was accompanied by significant changes in the associated global EV-proteome remained unaddressed. Label-free MS-based proteomic analysis resulted in evidence for a divergent EV-associated proteome between macrophage subtypes (Fig. 6, Supplementary Figure S10). Remarkably, the number of protein groups (protein IDs) identified in the resulting dataset differed significantly between subtypes (Supplementary Figure S10A, Supplementary Table S5), with M2-like macrophages displaying the least complex proteome (*n* = 1,435) followed by TAMs and M1-like cells, which showed a higher complexity (*n* = 2,157 and 2,771, respectively) (Fig. 6A). Principal Component Analysis (PCA) (Supplementary Figure S10B) and correlation analyses (Supplementary Figure S10C) confirmed consistency between biological replicates and clear separation of the EV samples according to macrophage subtype. None the less, a “macrophage EV-core” set of associated proteins (*n* = 672 protein IDs) was detectable irrespective of the macrophage subtype (Supplementary Figure S10D, Supplementary Table S6).

Prior to dissecting proteins differentially abundant in macrophage-derived EVs, we tested whether the relative contribution of diverse EV-related components was quantifiable from our bulk proteomic data. Inspired by the MISEV2023 guidelines (Welsh et al. 2024), a total of 1,952 human gene-encoded proteins were annotated and classified into corresponding (sub-)categories (Supplementary Table S4). Main categories included: (1) transmembrane proteins associated with plasma membrane/endosomes; (2) cytosolic proteins typically associated to EVs; (3) major non-EV co-isolated structures (lipoproteins, exomeres, supermeres); (4) proteins associated with intracellular compartments other than plasma membrane/endosomes; and (5) secreted and/or corona proteins. In total, 504 proteins of our merged macrophage EV proteome matched this classification (Supplementary Table S7), with marked differences among macrophage subtypes (Fig. 6A), as expected from the differences in the global EV-proteome complexity detected. When analyzing the relative abundance of the main MISEV categories as a whole (based on the number of precursors detected, Np), significant differences were also observed, especially in proteins belonging to categories 1 (transmembrane) and 2 (cytosolic), but also categories 4 (organelle-associated) and 5 (secreted/corona) (Fig. 6B). Proteins from categories 1 and 2 (typically associated with EVs) were significantly more prevalent in M2-like EVs as compared to M1- or TAM-like samples. This was especially evident for single-pass transmembrane proteins as Major Histocompatibility Complexes (MHC-I and MHC-II), which belong to category 1b, and members of the ESCRT complex, which are part of category 2a (Supplementary Figure S11). Altogether, these results supported higher endosomal contribution (around 1.5-fold) in M2-like EVs in comparison to M1- or TAM-like EVs (Fig. 6C). Of note, the overall analysis of category 3 (non-EV components and/or contaminants) did not show significant changes between macrophage-EVs (Fig. 6B), hinting at comparable levels of cross-contamination. Interestingly, M1-like EVs showed a significant higher proportion of proteins annotated in category 4 (proteins associated with organelles and/or intracellular compartments other than endosomes). Further analysis indicated that M1-like EV proteins were significantly enriched in mitochondrial components (*e.g.,* FASN, fatty acid synthase, or VDAC2, outer mitochondrial membrane protein porin 2), as compared to both M2- and TAM-like EVs (Fig. 6D, Supplementary Figure S11). In line with these observations, a higher abundance of markers described to be enriched in larger EVs (*e.g.,* Mitofilin or PD-L1, Programmed cell death 1 ligand 1) (Schone et al. 2024) was also observed (Fig. 6E). Finally, TAM-like EVs showed a higher proportion of category 5 proteins (functional component of EVs) in comparison to both M1- and M2-like EVs (Fig. 6B). These changes were mainly attributed to corona-related (category 5a) and adhesion and extracellular matrix (ECM) proteins (category 5c) (Fig. 6F, Supplementary Figure S11). Recent studies revealed an important intracellular crosstalk between autophagy and mitochondria (Gong et al. 2024) and exosome release (Zubkova et al. 2024). When checking the relative abundance of autophagy-related markers in our EV samples, we could observe a general higher abundance in M1-EV samples in comparison to M2-EVs (Supplementary Figure S12A), including key regulators like OPTN (Optineurin) or ATG7 (Autophagy-related protein 7) (Supplementary Figure S12B). TAM-EVs showed divergent patterns (Supplementary Figure S12A), with high levels of proteins like ATG3 (Autophagy-related protein 3) or GORASP2 (Golgi reassembly-stacking protein 2), and low levels of others RAB35 (Ras-related protein Rab-35) or STX3 (Syntaxin 3), among others (Supplementary Figure S12B).

In summary, our data supported that classically activated macrophages, here represented by M1-like MDMs, have a more complex EV proteome, reflected by the abundance of mitochondrial components in the EVs, while TAM-like EVs were found to harbor a proteome combining M1/M2-like characteristics, with a significant enrichment of a putative protein corona. Furthermore, our proteomic data supported elevated release of small EVs (endosomal origin) by M2-like MDMs. These observations may explain the larger size of M1- and TAM-like EVs in contrast to M2-EVs, and support similar release levels for M1- and M2-like MDMs with differential contribution of mitochondrial and endosomal-origin vesicles, respectively. The analysis of autophagy markers on EVs revealed that differential regulation of secretory autophagy may be a potential mechanism behind these observations, also with relevance in TAM-like MDMs. Additionally, functionally relevant corona-associated proteins were more prominent in TAM-like EVs, which may hint at enhanced immunomodulatory potency, possibly counteracting the relative low particle release observed for these cells.

### TAM-derived EVs carry a distinctive proteome with relevant prognostic value

**Fig. 7:**
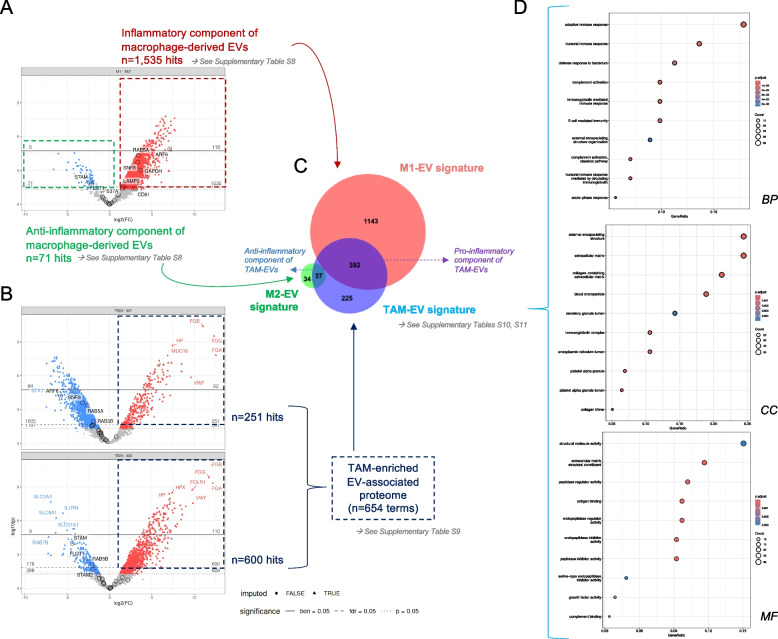
Signatures of the EV-associated proteome of macrophages. In this study, different contrasts were performed, allowing us to depict differential macrophage-EV signatures. Only differentially abundant proteins (DAPs) were considered (FDR-adjusted *p* value < 0.05). **A** Pro- and anti-inflammatory protein component of macrophage-derived EVs. Volcano-plot showing the comparative analysis of M1-like and M2-like samples. DAPs significantly more abundant in M1- or M2-like EVs defined the pro- and anti-inflammatory component of macrophage-derived EVs, respectively (*n* = 1,535 and *n* = 71 hits, see Supplementary Table S8 for details). B TAM-enriched component of EVs (*n* = 654 hits). Volcano-plots showing the comparative analysis of TAM-like (red) *vs*. M1- (upper panel, blue) or *vs*. M2-like samples (lower panel, blue) (see Supplementary Table S9 for details). **C** Macrophage-EV protein signatures obtained from the differential studies. Venn diagram shows the unique and intersecting signatures of macrophage-EVs, assigned according to their subtype (M1-, M2- and TAM-EV signatures; and, pro- and anti-inflammatory component of TAM-EVs, respectively). For further details about protein lists, see Supplementary Table S10. (D) Functional enrichment of the TAM-EV signature. Bubble plots representing top-10 significantly enriched (adjusted *p* value < 0.05) *Gene Ontology* (GO) terms in the TAM-EV signature panel (*n* = 225 proteins) segregated by *Biological Process* (BP), *Cellular Component* (CC) or *Molecular Function* (MF). Additional terms found by over-representation analyses (ORA) are reported in Supplementary Table S11

After evaluating the phenotypic characteristics and the potential alteration of EV biogenesis affecting cargo sorting, TAM-like EVs were analyzed with respect to their clinical relevance. Our results showed TAM-EVs to fall between M1- and M2-derived EVs with respect to proteome complexity (Fig. 6A, Supplementary Figure S10A), as well as with respect to relative abundance of MISEV-annotated proteins (Fig. 6B, Supplementary Figure S11). It is known that TAMs display a mixed phenotype (Boutilier and Elsawa 2021; Reinartz et al. 2014), showing both pro- and anti-inflammatory characteristics, but to the best of our knowledge to which extent TAM-EVs reflect this phenotype remains to be addressed. To explore this question, differentially abundant proteins (DAPs) (FDR-adjusted *p* value < 0.05) among macrophage-EVs were evaluated. M1- and M2-like EVs comparison defined the pro- (*n* = 1,535) and anti-inflammatory (*n* = 71) component of macrophage-EVs, respectively (Fig. 7A, Supplementary Table S8). The comparison between TAM- and M1-like EVs, and M2-like EVs resulted in 251 and 600 DAPs, respectively (Fig. 7B, Supplementary Table S9), which allowed the definition of an enriched TAM-EV proteome (*n* = 654 proteins). The comparison between the TAM-enriched proteome with the pro- and anti-inflammatory EV-profiles yielded a “unique” TAM-EV signature, not intersecting with any of the other groups (*n* = 225 DAPs, Fig. 7C, Supplementary Table S10). Conversely, exclusive M1- and M2-EV signatures were also defined (*n* = 1,143 DAPs and *n* = 34 DAPs, respectively, Supplementary Table S10). Moreover, the intersecting proteins between TAM- and M1-like EVs and between TAM- and M2-like EVs were considered the “Pro-inflammatory component of TAM-EVs” (*n* = 392 DAPs, Supplementary Table S10) and the “Anti-inflammatory component of TAM-EVs” (*n* = 37 DAPs, Supplementary Table S10), respectively. It is noteworthy that this approach comprised 16 out of the 62 candidates for TAM-EVs previously proposed by Cianciaruso et al*.* (Cianciaruso et al. 2019) (Supplementary Table S10), while further dissecting the pro- and anti-inflammatory component of macrophage-derived EVs. Functional pathway analyses of the TAM-EV signature (*n* = 225 proteins) highlighted the significant enrichment of proteins related to the *adaptative immune response* (GO:0002250), *complement activation* (GO:0006956), *collagen-containing extracellular matrix* (GO:0062023), *growth factor activity* (GO:0008083), *antigen binging* (GO:0003823) or *cargo receptor activity* (GO:0038024), among others (Fig. 7D, Supplementary Table S11), suggesting a pro-tumorigenic role of TAMs through diverse EV-mediated mechanisms. Of note, the role of ascites-derived TAMs in ECM reorganization was previously highlighted by transcriptomic analyses (Finkernagel et al. 2016). Here, our results have confirmed that certain signature genes associated to ECM remodeling like *COL1A1* (Collagen alpha-1(I) chain) or *LUM* (Lumican) (Supplementary Table S11), are also reflected at the secretome level as part of the TAM-EV protein signature (Supplementary Table S10).

**Fig. 8:**
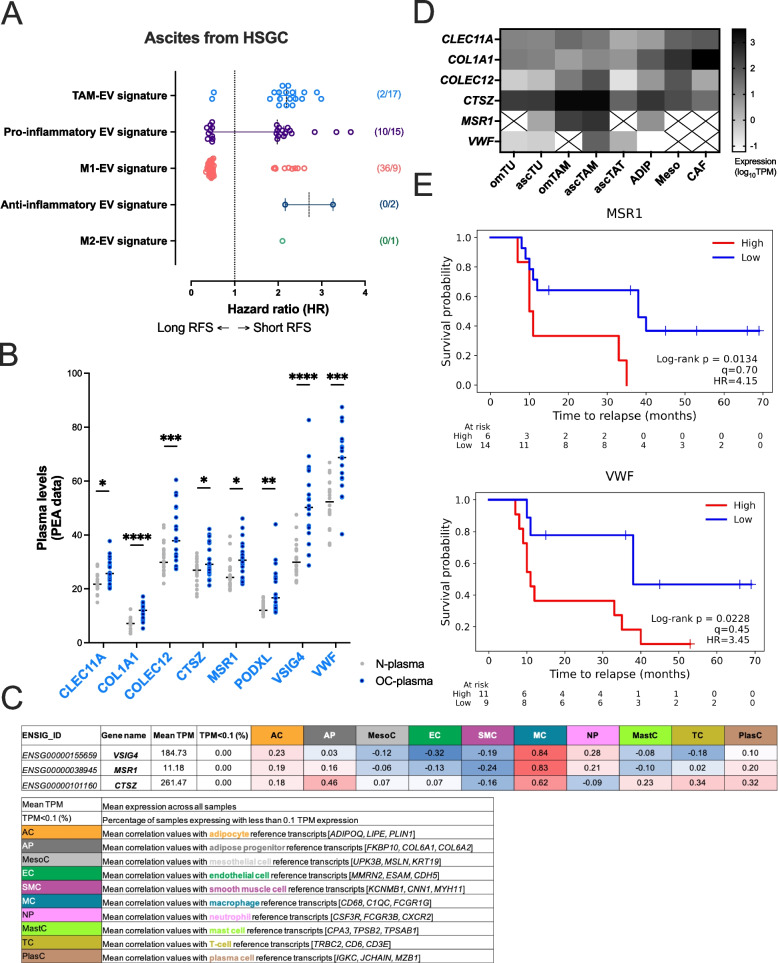
The EV-associated proteome of TAMs has unique traits with clinical value in OC. **A** Protein signatures found in macrophage-EVs with prognostic value in cell-free ascites from HGSC patients. Scatter-dot plots show the hazard ratio (HR) of the individual proteins significantly associated with long (HR < 1) or short (HR > 1) relapse-free survival (RFS) (Finkernagel et al, 2019). Median with 95% CI of the group is indicated. **B** Relative protein levels of TAM-EV signature markers showing significant differences in plasma from HGSC patients (*n* = 20; OC-plasma, blue), and patients with benign gynecologic diseases (*n* = 10; N-plasma, gray) as previously determined by PEA (Olink) Technology (Graumann et al, 2019). **C** Expression levels of TAM-EV markers in the human adipose tissue cell-type atlas (Norreen-Thorsen et al, 2022). Only macrophage-enriched markers are indicated. The table includes the mean TPM (transcript per million), detectability (TPM > 0.1%), and a heatmap of pairwise Spearman correlation coefficients for the specific markers and reference expression values in different resident cells of visceral adipose tissue (females), as described in the legend below. **D** Heatmap showing the expression levels (RNA-Seq adjusted, log_10_TPM median values) of the TAM-EV signature with clinical value across different cells of the OC TME (Sommerfeld et al, 2021). Only genes with TPM > 3 are shown. **E** Association of MSR1 and VWF levels in plasma with the survival of OC patients. Kaplan–Meier plots show the corresponding results for *n* = 20 HGSC patients analyzed by SomaScan technology (Graumann et al, 2019). Red and blue lines indicate high or low abundant levels of selected proteins, respectively. *q* indicates the quantile used for splitting datasets. HR, hazard-ratio; Om, *omentum*; asc, ascites fluid; TU, tumor cells; TAM, tumor-associated macrophages; TAT, tumor-associated T cells; ADIP, adipocytes; Meso, mesothelial cells; CAF, cancer-associated fibroblasts

Finally, we evaluated the clinical relevance of the macrophage EV-signatures established in this work (Fig. 8). To this end, the correlation between the relapse-free survival (RFS) of HGSC patients and the abundance of signature proteins was analyzed in a previous SomaScan dataset (Fig. 8A, Supplementary Table S10), studying the correlation of RFS to protein abundance in ascites fluid of HGSC patients (Finkernagel et al. 2019). We focused exclusively on proteins showing significant association to RFS, using Hazard Ratios (HR) as prognostic read-out. This analysis revealed, on the one hand, that a majority of or even all of proteins related to TAM-, M2-, and anti-inflammatory-signature of TAMs were related to a short RFS (HR > 1), implying bad prognosis. Signature proteins included CD163 or TNC (Tenascin) (Supplementary Table S8), which were previously related to poor prognosis in OC patients (Steitz et al. 2020; Reinartz et al. 2014; Finkernagel et al. 2019). In contrast, the majority of proteins in the M1-like signature correlated with a longer RFS (HR < 1) (Fig. 8A), and the pro-inflammatory signature of TAM-EVs exhibited proteins correlating with both long and short RFS. The detection of MRC1 (also known as CD206) in TAM-EVs in this context (related to short RFS) replicated work from the literature (Vyhlidalova Kotrbova et al. 2024; Cianciaruso et al. 2019). Altogether, our results provide new evidence for exacerbated worsening of patient-outcome by immunosuppression, here represented by signatures of M2- and TAM-EVs, respectively.

Finally, we checked whether components of the protein TAM-EV signature were detectable at circulating levels despite the low EV release of TAMs. By examining our previous data from high-sensitive Proximity Extension Assay (PEA) (Graumann et al. 2019), we confirmed the detection of 52 markers of the TAM-EV signature, 8 of which showed significant increased levels in the plasma of OC patients *vs*. non-cancer patients (*n* = 20/20) (CLEC11A, COL1A1, COLEC12, CTSZ, MSR1, PODXL, VSIG4, VWF) (Fig. 8B, Supplementary Table S10). Except PODXL (Podocalyxin), all proteins from this panel were representative of the most significant pro-tumorigenic functions of TAM-EVs (Supplementary Table S11). *VSIG4* (V-set and immunoglobulin domain-containing protein 4), *MSR1* (Macrophage scavenger receptor types I and II, also known as CD204) and *CTSZ* (Cathepsin Z) gene expression was found to be significantly enriched in macrophages from visceral adipose tissue of healthy females (Norreen-Thorsen et al. 2022) (Fig. 8C). Likewise, *COLEC12* (Collectin-12), *CTSZ*, *MSR1* and *VWF* (von Willebrand factor) corresponding transcripts were found to be highly expressed in omental and ascites TAMs in comparison to other host cells of the OC TME (Sommerfeld et al. 2021) (Fig. 8D). When exploring the prognostic significance of these macrophage-relevant candidates, our data confirmed a shorter RFS for OC patients with high plasma levels of MSR1 (10.5 *vs*. 37 months), as well as VWF (11 *vs*. 38 months), respectively (Log-rank *p* value < 0.05, Fig. 8E). Although our patients did not receive any therapy prior to ascites or plasma sample collection, maintenance treatment based on Avastin® (bevacizumab) could potentially influence our results (Monk et al. 2025). Avastin® therapy did not show significant associations to RFS in either our ascites (Supplementary Figure S13A) or plasma cohorts (Supplementary Figure S13B). When addressing RFS associations in treated *vs*. non-treated patients applying the same splitting thresholds, both MSR1 and VWF markers showed similar results (Supplementary Figure S13C), further supporting the diagnostic and prognostic value of the TAM-EV signature here identified.

## Discussion

The secretory profile of TAMs as compared to other – classical – macrophage subtypes has been addressed in previous works (Steitz et al. 2020; Puttock et al. 2023; Karagiannis et al. 2010; Cruz et al. 2024), emphasizing their proinflammatory character in the context of OC. Among others, TAMs are important producers of cytokines like IL-6/10, CCL18/22, TNF-α or TGF-β which are known to support cancer metastasis, adhesion, invasion and proliferation of tumor cells as well as chemoresistance (Worzfeld et al. 2018; Rodriguez et al. 2001; Dijkgraaf et al. 2013; Liu et al. 2020). In contrast to well-recognized soluble factors, little is known about the contribution of TAM-derived EVs, in general and in OC in particular. A recent study on ascites-derived vesicles highlighted significant contribution of macrophages to the EV-landscape of the OC TME (Vyhlidalova Kotrbova et al. 2024), though full depictions on their EV-associated proteome were missing. With the exception of a small number of studies, a majority of work has focused on M2-EVs as alternate models for TAM-EVs (reviewed in Zhou et al. 2023b; Liu et al. 2025a) and recent comprehensive comparative studies for M1- and M2-derived EVs excluded TAMs from the experimental conditions (Dechantsreiter et al. 2022), limiting the extension of their conclusions to OC. In order to address this gap in our knowledge, we collected and extensively characterized small EVs derived from M1-, M2- and ascites polarized TAMs.

First, the characterization of particle release at different levels during the preparatory workflow (conditioned media, EV-enriched and EV pelleted samples), led us to conclude that small EV release is reduced in TAMs, as compared to M1- or M2-like macrophages, displaying also differences in size and tetraspanin subpopulations. Our results differ from previous studies, where, for example, Pantazi et al*.* showed an increased release of EVs in M1 macrophages in comparison to M2, but no differences in median size (Pantazi et al. 2024). Another study investigating EVs derived from non-polarized macrophages in comparison to melanoma TAMs found a significantly increase of EV release in TAMs (Zhong et al. 2023). Similarly, primary isolated TAMs from human OC tissue showed higher EV release in comparison to THP-1 (monocyte macrophage cell line) (Lu et al. 2021). These discrepancies could be explained not only by the different models for macrophage polarization (Boutilier and Elsawa 2021; Mantovani et al. 2013) or technical approaches for EV collection and characterization (Lucotti et al. 2022), but also by the intrinsic effect of the surrounding microenvironment on macrophages, represented in this work by ascites fluid in contrast to, for example, the primary tumor site (Lu et al. 2021). It is likely that TAMs experience nutrient-challenged and metabolically stressed conditions within the ascites TME (reviewed in Kumar et al., 2022), which may shift these macrophages away from classical EV biogenesis toward alternative or unconventional secretory pathways (Rabouille 2017). In line with these hypotheses, we observed significant differences in EV-tetraspanin subpopulations between macrophages, with a particular low percentage observed in TAM-derived EVs. EVs are a heterogeneous population, harboring EVs originating from different biogenesis routes (Buzas 2023). Commonly used approaches for EV characterization rely on canonical markers such as tetraspanins (*e.g.,* CD9, CD63 or CD81) to evaluate the purity and/or efficacy of isolation (Welsh et al. 2024). These methods may, however, bias the characterization of EV preparations towards subpopulations resulting from specific biogenic pathways (Jeppesen et al. 2023; Niel et al. 2018). Looking at CD9 + vesicles, the frequency did not show significant changes between M1- and M2-like but a significantly reduced frequency for TAM-EVs. Additionally, the size profile of CD9 + EVs also supported a smaller size already detected in the total EV population of M2-cells, while M1- and TAM-like size EV-profiles yielded larger medians and size deviation. Also, CD63 + subpopulation indicated a significantly higher frequency in M2-like EVs, compared to both M1- and TAM-like EVs. As CD63 has been proposed as a marker for the endosomal pathway (Jeppesen et al. 2023), the observed frequency may point to an upregulated endosomal machinery in M2-like macrophages. Interestingly, the size profile of CD63 + EVs was comparable across macrophages, which further supports a common biogenesis route (most likely endosomal). The size profiles of CD9 + and CD81 + EVs were, in contrast, different between macrophage subtypes, suggesting these vesicles to have different or additional routes of origin. In this regard, CD9 has been suggested to be not only a marker for the endosomal pathway but also for membrane-budded particles (Mathieu et al. 2021), which could partially explain why M1- and M2- show comparable total EV release. Additionally, it has been shown that an overexpression of CD9 results in increased exosome release (Boker et al. 2018). In this regard, the higher frequency of CD9 + EVs in M1- and M2-like samples supported the correlation to a higher release rate, also in line with the observed low CD9 frequency and total EV release of TAM-like cells.

Our cellular analysis further supported alterations in EV release with potential influence on protein cargo loading in macrophages. For instance, at the cellular level we confirmed the alteration of EV-related markers like FLOT1, LAMP1 or CD63 among macrophages. FLOT1, together with other flotillins, has been shown to associate with membrane microdomains which are highly abundant in cholesterol and sphingolipids and involved in endocytosis, intracellular transport, and signaling (Pust et al. 2010). Interestingly, FLOT1 itself has been reported not to affect EV release but protein cargo (Phuyal et al. 2014), which may explain the proteomic differences observed between M1- and M2-EVs in contrast to TAM-EVs. Conversely, LAMP1 was significantly increased in TAM-like cells, which is present on the plasma membrane and organelles in the endo-lysosomal pathway, as well as degradative lysosomes (Cheng et al. 2018). Although the function of LAMP1 in EV formation and release is context-dependent (Alessandrini et al. 2017), our data may indicate that multi-vesicular bodies (MVBs) of TAMs are more prone to degradation than fusion/release. Our proteomic data further supported these potential mechanisms, as proteins involved in MVB fusion from the SNARE (Soluble N-ethylmaleimide-sensitive factor attachment protein receptors) complex such as STX3, STX7 (Syntaxin 7) or SNAP29 (Synaptosomal-associated protein 29) (Liu et al. 2024), were particularly low in TAM-like EV samples. Also remarkably, high expression of LAMP1 was determined as a prognostic marker for OC patients, correlating with a poor outcome (Xu et al. 2017), and corroborating its pro-tumorigenic role in TAMs.

Furthermore, one study revealed a reduction of particle release upon CD63 knockdown (Hurwitz et al. 2016). Our data support these observations for macrophages, as cellular protein levels reflect CD63 + EV-subpopulation frequency. A parallel observation for CD63 in our model was that the levels of this protein on macrophage-EVs seemed to be highly influenced by the N-glycosylation status of the protein. In fact, it was striking to detect that the typical smeary pattern of CD63 shifted to a lower molecular weight more predominantly in TAMs, pointing to differences in its PTM status. To the best of our knowledge, this study is the first to report a differential CD63 N-glycosylation at the cellular level in TAMs. In addition to its role in EV-biogenesis, high CD63 expression in breast cancer or melanoma has been associated with increased invasive capacity and metastatic potential of tumor cells, likely through interactions with integrins or metalloproteinases (Hemler 2014). It can be hypothesized that decreased CD63 N-glycosylation in TAMs may influence cell–cell or cell–matrix interactions, altering their immunomodulatory effects and/or their invasion capacity, opening new avenues to address in the future. Importantly, we validated the underlying observations in patient-derived samples, highlighting that our established in vitro system (Steitz et al. 2020; Sommerfeld et al. 2022) is a suitable model for EV-related studies in OC, particularly ascites-derived TAMs. Moreover, our data indicated that higher N-glycosylated form was predominantly found in all macrophage-EVs, pointing to a role of this PTM regarding CD63 packaging. As CD63 was generally less abundant on EVs compared to cells, and TAMs in particular displayed lower N-Glycosylation levels, it may be speculated that the higher N-glycosylated form is preferentially loaded in macrophage-EVs, while both forms can be present in the cells. The importance of glycosylation for EV packaging has been previously reported for AZU (Azurocidin) (Naito et al. 2021) and TROP2 (Trophoblast antigen 2) (Kamble et al. 2021). Whether the potential role of N-glycosylation of CD63 is only relevant in macrophages and/or limited to EV-sorting needs to be further investigated.

In order to get a more comprehensive overview of macrophage-derived EVs, we further investigated the associated proteomes. Based on the number of detected proteins, M1-like EVs exhibited the most complex proteome, while M2-like EVs displayed significantly reduced complexity, placing TAM-like EVs in an intermediate position. To gain further insight, we performed a novel analysis dissecting our bulk EV-proteome data according to MISEV categories (Welsh et al. 2024). On the one hand, we found that M2-like EVs showed a significantly higher proportion of proteins belonging to categories 1 and 2 in comparison to M1- and TAM-like EVs, encompassing transmembrane proteins associated with plasma membrane and/or endosomes, as well as cytosolic proteins in EVs (Welsh et al. 2024). These results were in line with the higher EV release observed for M2 macrophages, likely related to endosomal-related proteins. On the other hand, M1-like EVs indicated a significantly higher proportion of category 4 proteins including those associated with intracellular compartments other than endosomes, especially mitochondrial components. Particularly, members of the ATP synthase complex and FASN, as well as VDAC2, were significantly more abundant on M1-like EVs. Our data support a potential role of the pro-inflammatory stimuli in a more abundant packing of mitochondrial-related proteins, or even point to a potential contribution of “mitoEVs” in macrophages. It has been described that especially large EVs from M1 and M2 macrophages carry mitochondria or mitochondrial fragments (Hua et al. 2024), as well as mitochondrial DNA (Gao et al. 2024). Although our isolation protocol was intended to study small EVs, we evaluated the abundance of EV markers previously associated to ectosomes or large EVs (Schone et al. 2024). In general, we found a higher abundance of these markers (like PD-L1 or Mitofilin) in M1-like preparations, supporting a potential contribution of larger particles in M1-EVs and explaining the significant relevance of mitochondrial components in these EVs. As for the high abundance of autophagy-related factors found in M1-EV samples (Zubkova et al. 2024), our data suggest that secretory autophagy could (at least partially) mediate the secretion of these mitochondrial components upon inflammatory stimuli in macrophages. Whether these observations respond to specific packaging or disposal of “damaged” mitochondrial components (Gong et al. 2024), guarantees further investigation.

Finally, TAM-like EVs indicated a higher proportion of glycoproteins like MUC1 (Mucin-1, also known as CD227) and other functionally relevant proteins, suggesting a more extensive putative EV corona as in comparison to M1- and M2-like EVs, further pointing to different functionality of TAM-EVs. Notably, MUC1 has previously been correlated to OC progression (Wang et al. 2007) and adhesion proteins play an important role in the uptake of EVs by the recipient cell (Altei et al. 2020). We hypothesize TAM-EVs could therefore modulate their effects to target cells in a non-particle-dependent manner. These inferences should, however, be considered with a grain of salt, as the existence and composition of an EV-corona remains controversial (Escudero-Cernuda et al. 2025; Dietz et al. 2023; Toth et al. 2021).

Lastly, we investigated how far the EV-associated proteome of TAMs in OC reflects the cells´ double-edged character combining both pro- and antitumorigenic potential (Liu et al. 2025a). Our data showed that TAM-EVs presented with both M1, as well as M2 characteristics at the EV level, but also a unique TAM-EV proteome. Our results extend previous studies on bulk ascites-derived EVs (Vyhlidalova Kotrbova et al. 2024; Quiralte et al. 2024) and primary spheroid-derived EVs (Christian et al. 2025), as we were able to correlate the macrophage EV-signatures with RFS of OC patients. More concretely, they suggested a link of the anti-inflammatory or immunomodulatory EV-signatures with aggravated survival in HGSC patients, highlighting the pro-tumorigenic role of TAMs also with respect to their EVs. On the other hand, the M1-EV and the pro-inflammatory TAM-EV signature shared proteins correlating with both longer and shorter RFS, recapitulating controversial observations in the literature (Cianciaruso et al. 2019). Finally, we investigated whether TAM-EV proteins harbor diagnostic and/or prognostic value at circulating levels by correlating the specific TAM-EV proteome to previous affinity proteomics datasets analyzing the plasma of OC and benign patients (Graumann et al. 2019). The resulting correlation revealed 8 proteins (CLEC11A, COL1A1, COLEC12, CTSZ, MSR1, PODXL, VSIG4, VWF) significantly enriched in OC patient plasma samples with particular high expression in TAMs in the OC TME (both ascites and omental). Remarkably, most of these proteins were involved in significantly enriched pro-tumorigenic processes of the TAM-EV signature, including, but not limited to, *complement activation*, *extracellular matrix remodeling* or *receptor binding activity*. One of the TAM-EV proteins associated to *complement binding* and *complement activation* pathways was VSIG4. Recent studies have highlighted the relevance of VSIG4 + macrophages in immunomodulatory responses in the tumor context (Pan et al. 2025, 2023). Based on these studies, we speculate that TAM-EVs could, for example, modulate neutrophil infiltration and impair antigen-specific T cell immunity through VSIG4 interaction (Pan et al. 2025). As previously indicated, our panel highlighted the enrichment of TAM-EVs in ECM proteins like COL1A1, COLEC12 or CTSZ. The role of TAMs in ECM reorganization in HGSC has been previously highlighted (Finkernagel et al. 2016), thus our data extends this functional relevance to their derived EVs. COL1A1 has been shown to facilitate metastasis in OC (Li et al. 2020a). For COLEC12, no data is available in the context of OC, but it has been described to be involved in the regulation of apoptosis and inflammation in osteosarcoma (Li et al. 2020b) and breast cancer metastasis (Elola et al. 2007). Proteins with *receptor binding activity* in the TAM-EV signature included CLEC11A (C-type lectin domain family 11 member A), among others. CLEC11A (also known as SCGF) is a functional glycoprotein fundamentally involved in hematopoietic differentiation (Wang et al. 2020b). CLEC11A plasma levels have been recently reported to correlate with an elevated risk of OC (Liu et al. 2025b), although the mechanisms governing its cancer-promoting effects are still poorly understood (reviewed in Wang et al. 2020b).

Furthermore, we found plasma levels of MSR1 (or CD204) and VWF, to correlate with shorter RFS in OC patients. MSR1 is scavenger receptor found on the surface of various types of macrophages, presenting a dichotomic role either protective or detrimental to the pathogenesis of disease (reviewed in Gudgeon et al. (2022)). MSR1 has been found to promote OC and pancreatic cancer invasion and metastasis (Neyen et al. 2013). More recently, a high infiltration of MSR1 + macrophages have been associated with lymph node metastasis in renal carcinoma (Xie et al. 2024), with particular increase of T cell exhaustion and upregulation of Treg subpopulation. Based on these evidences, our data suggest that TAM-EVs may promote modulatory effects of T cell subpopulations promoting OC progression and metastasis. In the case of VWF, several studies indicated a role in tumor cell adhesion in gastric cancer (Yang et al. 2018) and breast cancer (Gomes et al. 2005). Additionally, VWF has been shown to upregulate angiogenesis in breast cancer (Tao et al. 2022) and correlates with increased tumor vessel density in melanoma (Goertz et al. 2016). Mechanistically, a recent study has demonstrated a key modulatory role of VWF in EV-mediated angiogenesis in the context of hepatocellular carcinoma (Wong et al. 2023). The only exception for representing significantly enriched functions of the TAM-EV signature was PODXL. Interestingly, PODXL has been proposed as a key EV-associated protein involved in fibroblast activation with subsequent deposition of a highly pro-invasive ECM (Novo et al. 2018). Moreover, a recent study has shown that PODXL favors HGSC progression by protecting tumor spheroids from NK cell cytotoxicity (Tran et al. 2025). The potential functional implications of TAM-EVs could, thus, target different cells of the tumor stroma.

Altogether, our data demonstrates the study of TAM-derived EVs in OC as a valuable source for biomarker discovery, providing new candidate proteins whose mechanistic role in OC deserves further investigation.

### Study limitations

Our study provides new insights in the contribution of TAMs to the EV landscape of the HGSC TME; however, it entails some limitations. Our work is based on the study of primary samples (either peripheral blood monocytes or patient-derived TAMs, as well as ascites fluid) which strengthens the translational impact but, in turn, affects the scale of the experimental set-up. Particularly, the low EV release of TAM-like cells hampered the implementation of further purification protocols for EV preparations (*i.e.*, by size-exclusion chromatography). We have previously described technical biases when particle number input differs (Gómez-Serrano et al., 2021). Thus, in this work we favored the reliability of comparison (M1-EVs *vs*. M2-EVs *vs.* TAM-EVs) in detrimental of the purity of our samples (UC-isolated), which may be particularly relevant for proteomics. Although this constitutes a limitation, it allows to have more comparable data to recent references (see Cianciaruso et al. 2019; Quiralte et al. 2024), as they also relied on UC as isolation method. We cannot thus rule out the presence of co-isolates (*e.g.,* exomeres) (Tosar et al. 2022), nevertheless, the biological and translational relevance of these entities (refereed as NVEPs or simply EPs, extracellular particles) has been also recognized (reviewed in Lucotti et al. 2022). In this work, we refer to small EVs for simplicity but the presence of NVEPs may not be overlooked and deserves further investigation. As for our results, inclusion of the large EV fraction will be also of high interest in the study of the TAM-derived secretome.

Low EV-yield from TAM-like MDMs also hindered further mechanistic insights. Although we could speculate about potential implications based on the proteomic signature and previous literature findings, functional validation of the TAM-EV immunomodulatory capacity will be imperative in upcoming work. Future attempts may include pooling of monocyte donors or ex vivo cultured patient-derived TAMs as scaling-up options. Different target cells (including, but not limited to, T cells, fibroblasts or tumor cells) and EV-dose testing will also benefit future studies, especially if a more functional EV-corona is present on TAM-EVs in comparison to M1- or M2-EVs. In this regard, we hypothesize that immunomodulatory effects exerted by TAM-EVs may require less vesicles per cell than, for example, alternatively polarized M2-EVs to reach comparable effects. We also anticipate that whether a functional corona is more relevant in TAM-EVs, their functional evaluation in vitro may be affected by the isolation method of choice (Wolf et al. 2022).

In this work, we made an effort to evaluate the translational relevance of TAM-like MDMs as a surrogate model of ascites-derived TAMs for EV research. Our previous studies support the relevance of ascites-driven reprogramming of MDMs in helping to elucidate new mechanisms of OC progression by translating clinical findings to the in vitro setting (Steitz et al. 2020; Sommerfeld et al. 2022; Reinartz et al. 2014). In this study, we have observed that TAM-like MDMs recapitulated several features of patient-derived TAMs including, for example, transcriptional patterns on EV-biogenesis molecules, as well as reduced N-Glycosylation of cellular CD63 protein. Whether the TAM “EV-phenotype” is modulated by the soluble, EV-associated factors, or both, present in the ascites fluid deserves further investigation.

Finally, clinical heterogeneity is also a challenge in this research, which we tried to partially overcome with the use of ascites fluid pooled from different HGSC patients. Despite this constitutes an advantage for homogenization on the experimental and analytical flanks, it shortages the valuable information behind the individual characteristics. Correlation of clinical/individual variables with, for example, differential EV release, phenotype and cargo loading modulation in TAMs will foster the identification of new, more personalized markers.

## Conclusions and outlook

**Fig. 9:**
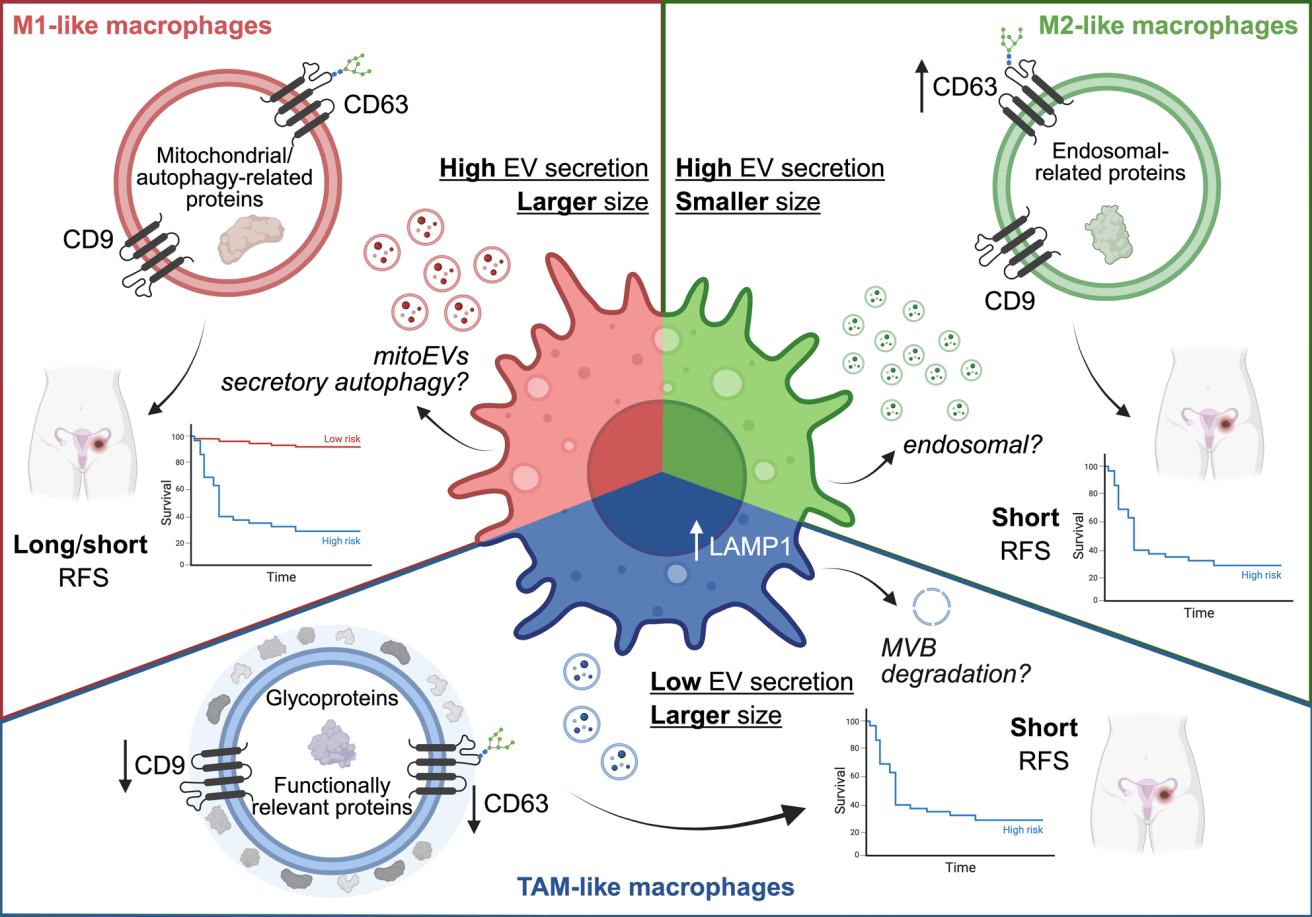
Macrophage reprogramming alters extracellular vesicle release and cargo composition. Our data indicate that ascites-driven reprogramming of monocytes into tumor-associated macrophages (TAMs) results in reduced extracellular vesicle (EV) release compared with M1- and M2-like macrophages. TAM-like cells display increased LAMP1 expression, indicative of enhanced lysosomal degradation. EVs derived from distinct macrophage subtypes (M1, M2, and TAM) exhibit differences in size distribution, frequency of tetraspanin-subpopulations (including CD9 and CD63), and differential cargo sorting (*i.e.,* mitochondrial and endosomal components). Altogether, these changes underscore differential cellular mechanisms modulating the macrophage-derived secretome. Interestingly, CD63 N-glycosylation levels are preserved in all macrophage-EVs, in contrast to cellular levels in TAMs. Proteomic profiling identified distinct protein signatures associated with M1-, M2-, and TAM-derived EVs, which display differential associations with ovarian cancer progression, as assessed by relapse-free survival (RFS). LAMP1, lysosome-associated membrane glycoprotein 1; MVB, multi-vesicular body. Created in BioRender: https://BioRender.com/sd5d4db

Our data confirmed ascites-reprogrammed TAMs to present specific EV release characteristics, reflected both at the cellular and the EV level (Fig. 9). Notably, small EV release is reduced in TAMs and EV tetraspanin-subpopulation sizes are closer to M1 than M2 cells, most likely resembling their pro-inflammatory character. Immunoblotting analyses of EV biogenesis-related markers demonstrated TAMs to harbor less FLOT1 (ESCRT-independent pathway) and more LAMP1 (lysosomal degradation) as well as to show decreased N-glycosylation status on CD63 protein in comparison to M1- or M2-like macrophages at the cellular level. We speculate that differential PTMs may influence the interaction of CD63 with other relevant proteins of the secretory machinery such as Rab GTPases (*e.g.,* Rab8A, Rab21A) or members of the SNARE family proteins (*e.g.,* syntaxins or VAMP proteins) (Cheerathodi et al. 2021). Tailored experiments (*e.g.,* immunoprecipitation assays) will help to elucidate the mechanisms behind the low EV release observed for TAM-like macrophages and whether the PTM status of CD63 influences these interactions. Interestingly, the differential N-glycosylation pattern of CD63 was not replicated at the EV-level in any of the macrophage subtypes. Deep proteomic characterization further led to the differential quantification of EV-protein cargo among macrophage subtypes (*e.g.,* autophagy-related, mitochondrial or endosomal components). Extending previous results (Cianciaruso et al. 2019), we further confirmed that the EV-associated proteome of TAM-like MDMs comprises unique traits, but also shares characteristics with both M1- or M2-like derived vesicles, thus reflecting the ambivalent character of these cells within the TME. Recently, TAM diversity in human cancers has been revealed by single-cell RNA-sequencing (scRNA-seq) (Wang et al. 2023), partially overcoming this M1/M2 dichotomy. Therefore, the potential co-existence of diverse TAM subtypes in the OC TME with different secretory profiles may be also a possibility, guaranteeing future investigation.

Finally, we showed that the EV-associated proteome of ascites TAM-like macrophages is enriched for factors like CD163, MRC1 or TNC that were previously related to poor prognosis in OC patients (Steitz et al. 2020; Reinartz et al. 2014; Finkernagel et al. 2019), and also for others like MSR1 or VWF, recapitulating findings for the soluble circulating proteins. The analyses of our cohorts (ascites, *n* = 70; plasma, *n* = 20 patients) did not show significant associations to RFS based on maintenance therapy (herein Avastin®). These effects may be, however, more prominent in larger cohorts and/or when in combination with other drugs (Monk et al. 2025), becoming a potential source of biases to be considered in follow-up studies. In conclusion, our results on TAM-EVs extend previous work studying the inflammatory tumor secretome of OC (Vyhlidalova Kotrbova et al. 2024; Steitz et al. 2020; Finkernagel et al. 2019; Quiralte et al. 2024; Christian et al. 2025), contributing to a better understanding on how macrophages integrate into the EV landscape of the TME.

## Supplementary Information

Supplementary Material 1: Supplementary Tables

Supplementary Material 2: Supplementary Figures

Supplementary Material 3: Supplementary Information

## Data Availability

Mass spectrometric raw data are available at the ProteomeXchange Consortium with dataset identifier: PXD065421, via the MassIVE partner repository (https://massive.ucsd.edu/, MassIVE ID: MSV000098313; doi:10.25345/C50V89W2H). Protein and annotation lists are also provided as Supplementary Information files.

## Abbreviations

ACN: Acetonitrile
ascMDM: Ascites-polarized monocyte-derived macrophage
ascTAM: Ascites-derived tumor-associated macrophage
ATG3: Autophagy-related protein 3
ATG7: Autophagy-related protein 7
AZU: Azurocidin
BCA: Bicinchoninic acid assay
BH: Benjamini–Hochberg
BP: Biological process
BSA: Bovine serum albumin
CTSZ: Cathepsin Z
CC: Cellular component
CCL18: C–C motif chemokine 18
CCL22: C–C motif chemokine 22
CLEC11A: C-type lectin domain family 11 member A
COL1A1: Collagen alpha-1(I) chain
COLEC12: Collectin-12
CD206: Macrophage mannose receptor 1, also known as MRC1
CD163: Scavenger receptor cysteine-rich type 1 protein M130
DIA: Data-independent mode
DAP: Differentially abundant protein
DTT: Dithiothreitol
EM: Electron microscopy
ECM: Extracellular matrix
EP: Extracellular particle
EV: Extracellular vesicle
FDR: False discovery rate
FASN: Fatty acid synthase
FBS: Fetal bovine serum
FA: Formic acid
FLOT1: Flotillin-1
GO: Gene ontology
GAPDH: Glyceraldehyde-3-phosphate dehydrogenase
GORASP2: Golgi reassembly-stacking protein 2
HR: Hazard ratio
HGSC: High-grade serous carcinoma
HPLC: High-performance liquid chromatography
ID: Identification
IL: Interleukin
IAA: Iodoacetamide
LRS: Leukocyte reduction system
LPS: Lipopolysaccharide
LUM: Lumican
LAMP1: Lysosome-associated membrane glycoprotein 1
MSR1: Macrophage scavenger receptor types I and II, also known as CD204
MS: Mass-spectrometry
MFI: Mean fluorescence intensity
MISEV: Minimal information for studies of extracellular vesicles
MF: Molecular function
MCP-1: Monocyte chemoattractant protein 1, also known as CCL2
MDM: Monocyte-derived macrophage
MUC1: Mucin-1, also known as CD227
MVB: Multivesicular body
nFC: Nano-flow cytometry
NVEP: Non-EV co-isolated structures
Np: Number of precursors
OPTN: Optineurin
OC: Ovarian cancer
ON: Over-night
ORA: Over-representation analysis
PFA: Paraformaldehyde
PBMC: Peripheral-blood mononuclear cell
PBS: Phosphate-buffered saline
PODXL: Podocalyxin
PTM: Post-translational modification
PCA: Principal component analysis
PD-L1: Programmed cell death 1 ligand 1
PEA: Proximity extension assay
RIPA: Radioimmunoprecipitation assay buffer
RSLC: Rapid separation liquid chromatography
RAB35: Ras-related protein Rab-35
RFS: Relapse-free survival
RT: Room temperature
SDS: Sodium dodecyl sulfate
SDS-PAGE: Sodium dodecyl sulfate polyacrylamide gel electrophoresis
SLS: Sodium lauroyl sarcosinate
SNARE: Soluble N-ethylmaleimide-sensitive factor attachment protein receptors
SNAP29: Synaptosomal-associated protein 29
STX3: Syntaxin 3
STX7: Syntaxin 7
TNC: Tenascin
TPS: Total protein staining
TBS: Tris-buffered saline
TBS-T: Tris-buffered saline with Tween-20
TEAB: Triethylammonium bicarbonate
TROP2: Trophoblast antigen 2
TAM: Tumor-associated macrophage
TME: Tumor microenvironment
TSG101: Tumor susceptibility gene 101 protein
UC: Ultracentrifugation
UF: Ultrafiltration
UT: Untreated
UKGM: University Hospital Gießen and Marburg
VDAC2: Outer mitochondrial membrane protein porin 2
VSIG4: V-set and immunoglobulin domain-containing protein 4
VWF: Von Willebrand factor
WHO: World Health Organization
