# Supplementary material for: Tumor-associated macrophages display differential protein cargo sorting in extracellular vesicles associated with poor survival in ovarian cancer
Source: Mol Med. 2026 Jan 30;32:16. doi: 10.1186/s10020-025-01416-x (PMC12875045; doi:10.1186/s10020-025-01416-x)
Supplement: Supplementary file 2 — Supplementary Material 2: Supplementary Figures [file 10020_2025_1416_MOESM2_ESM.pdf]

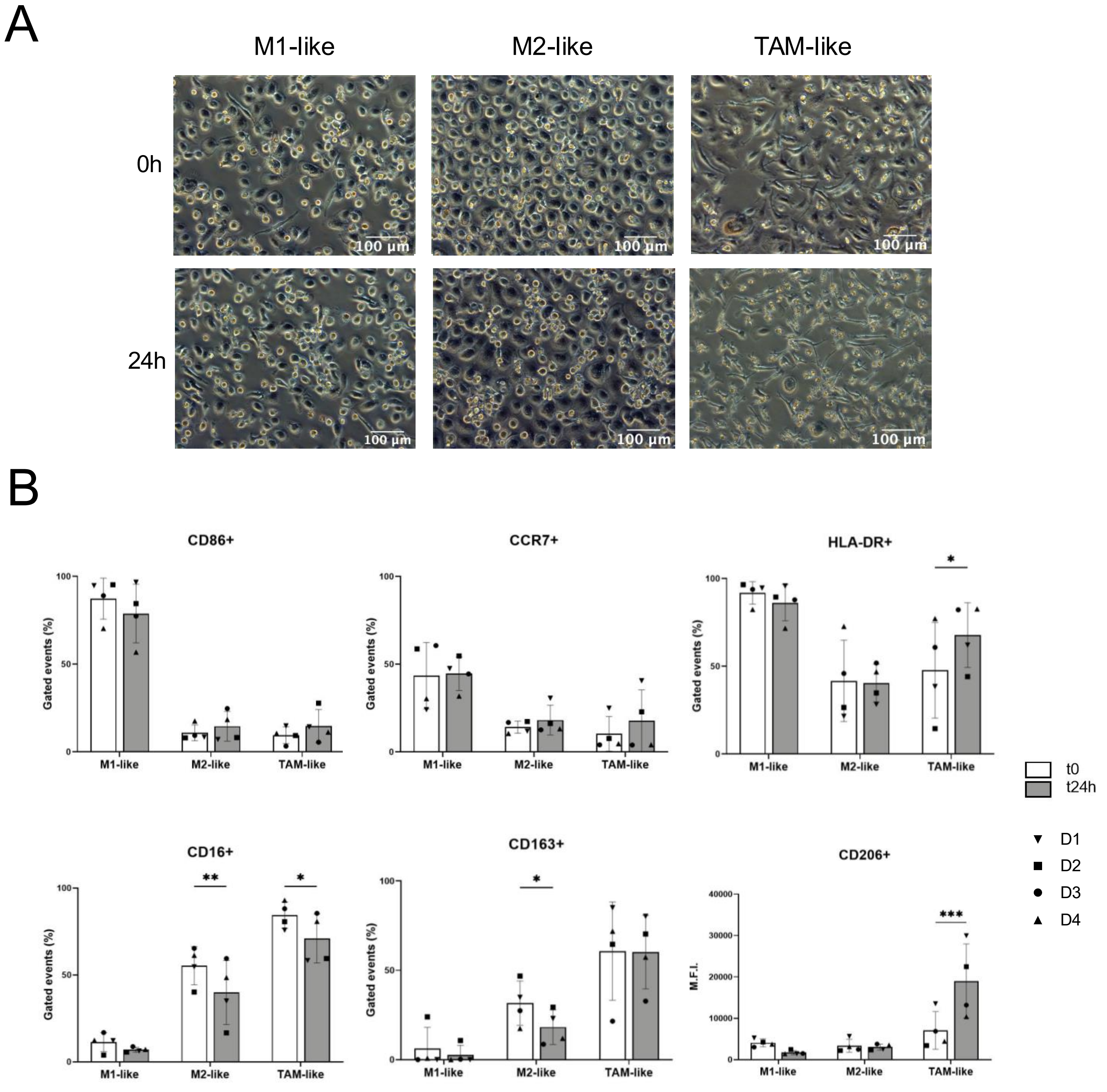


Figure S1
Pörschke *et al.*

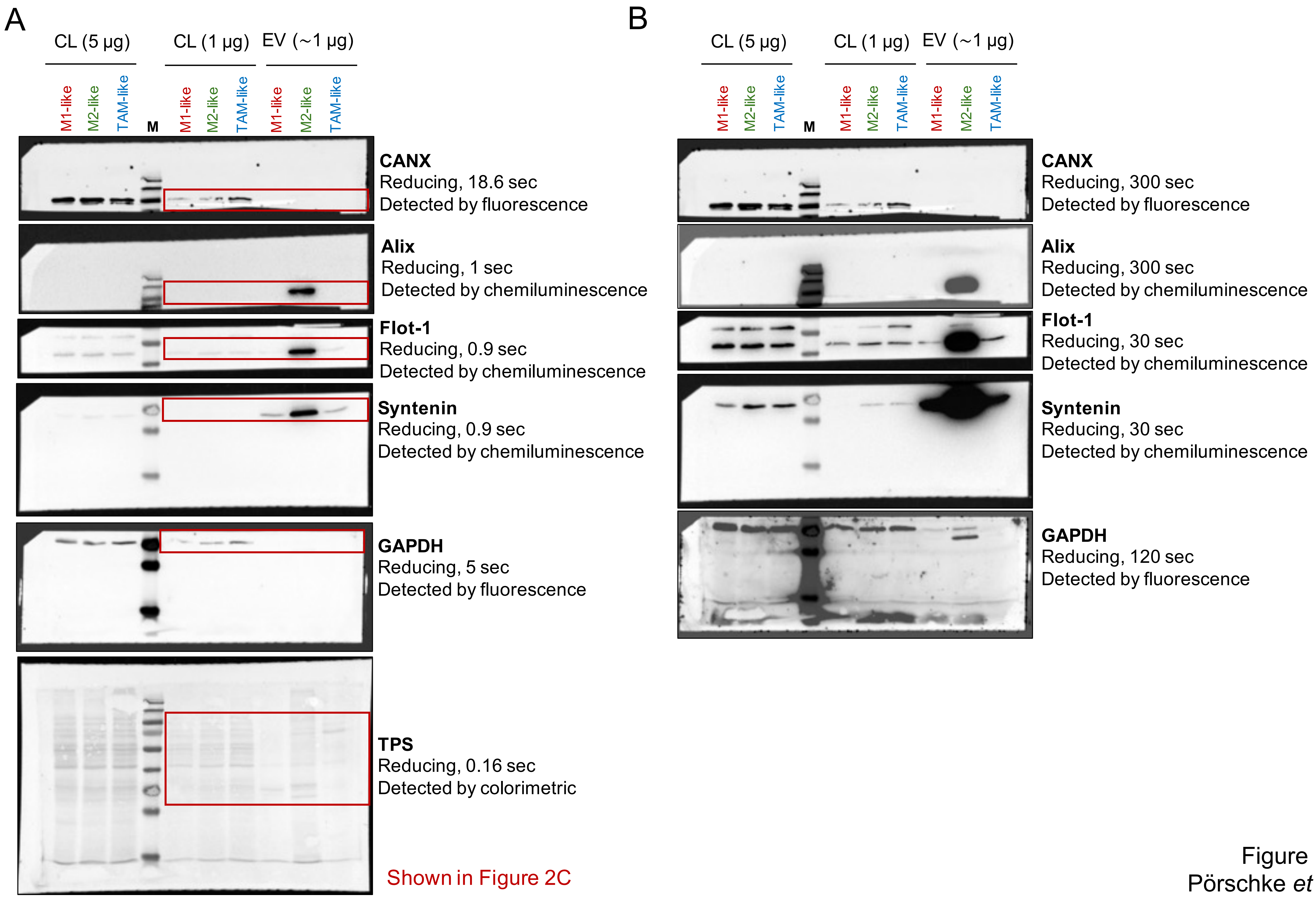


Figure S2
Pörschke *et al.*

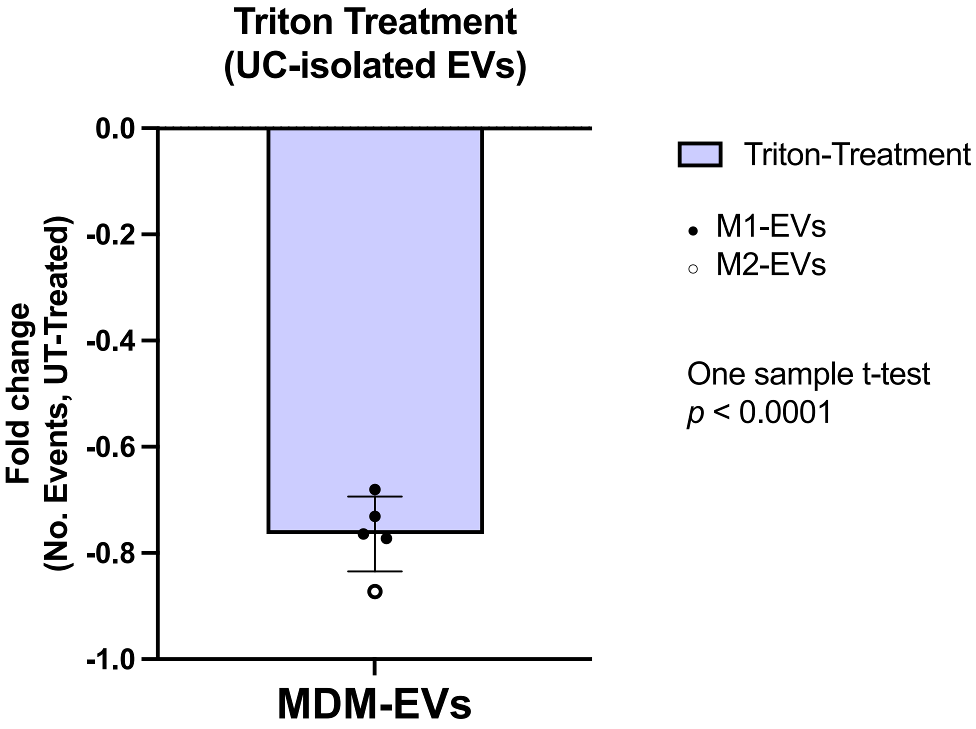


Figure S3
Pörschke *et al.*

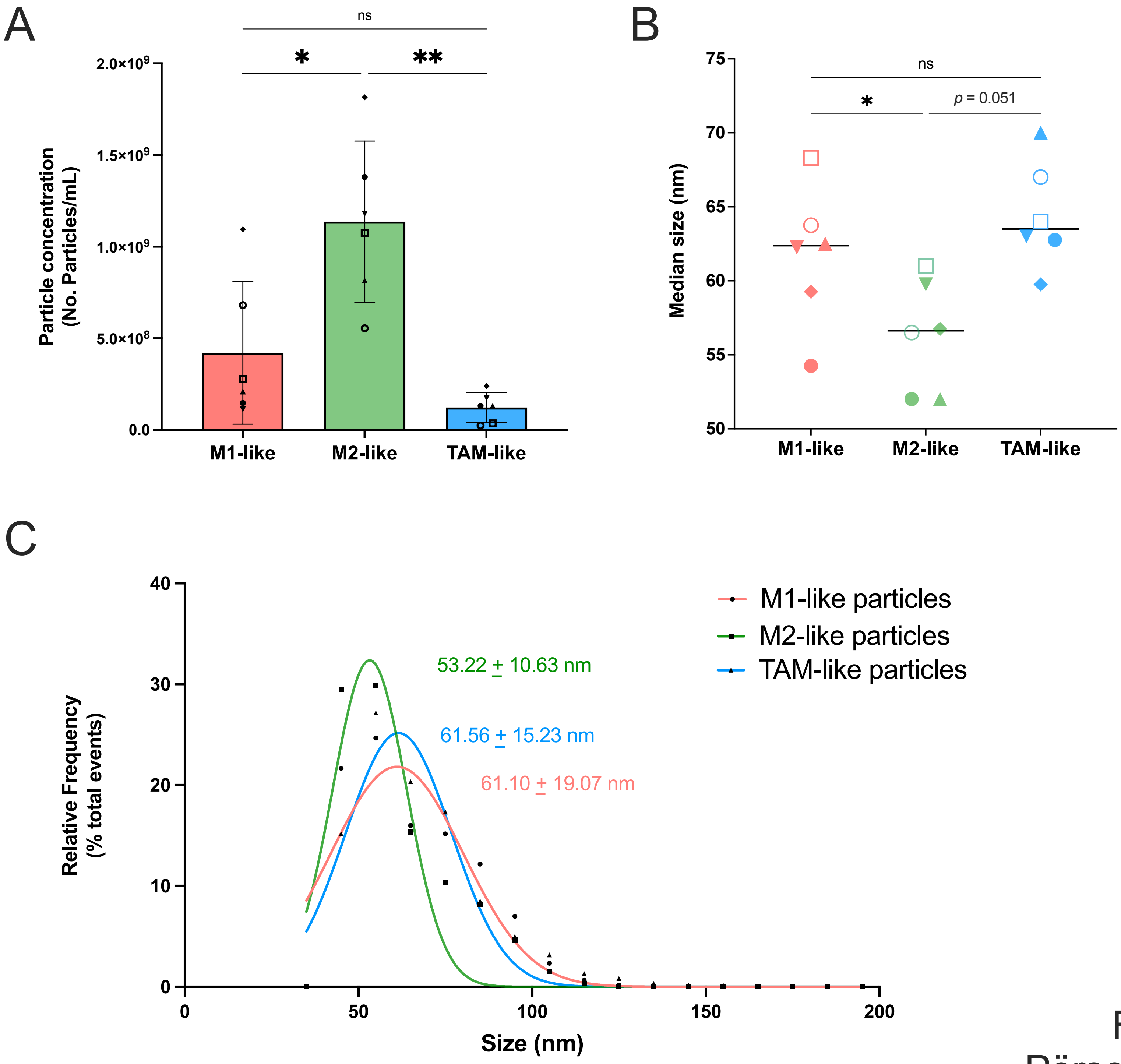


Figure S4
Pörschke *et al.*

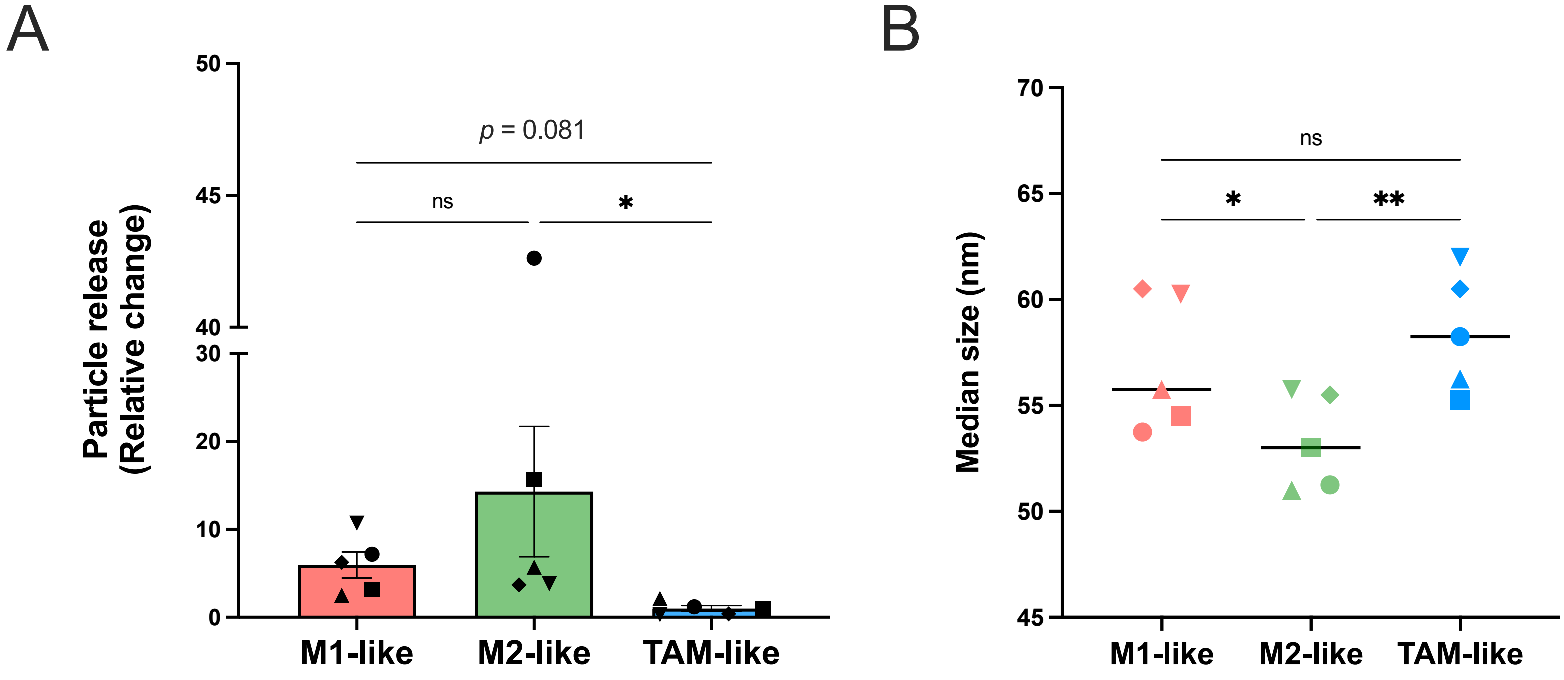


Figure S5
Pörschke *et al.*

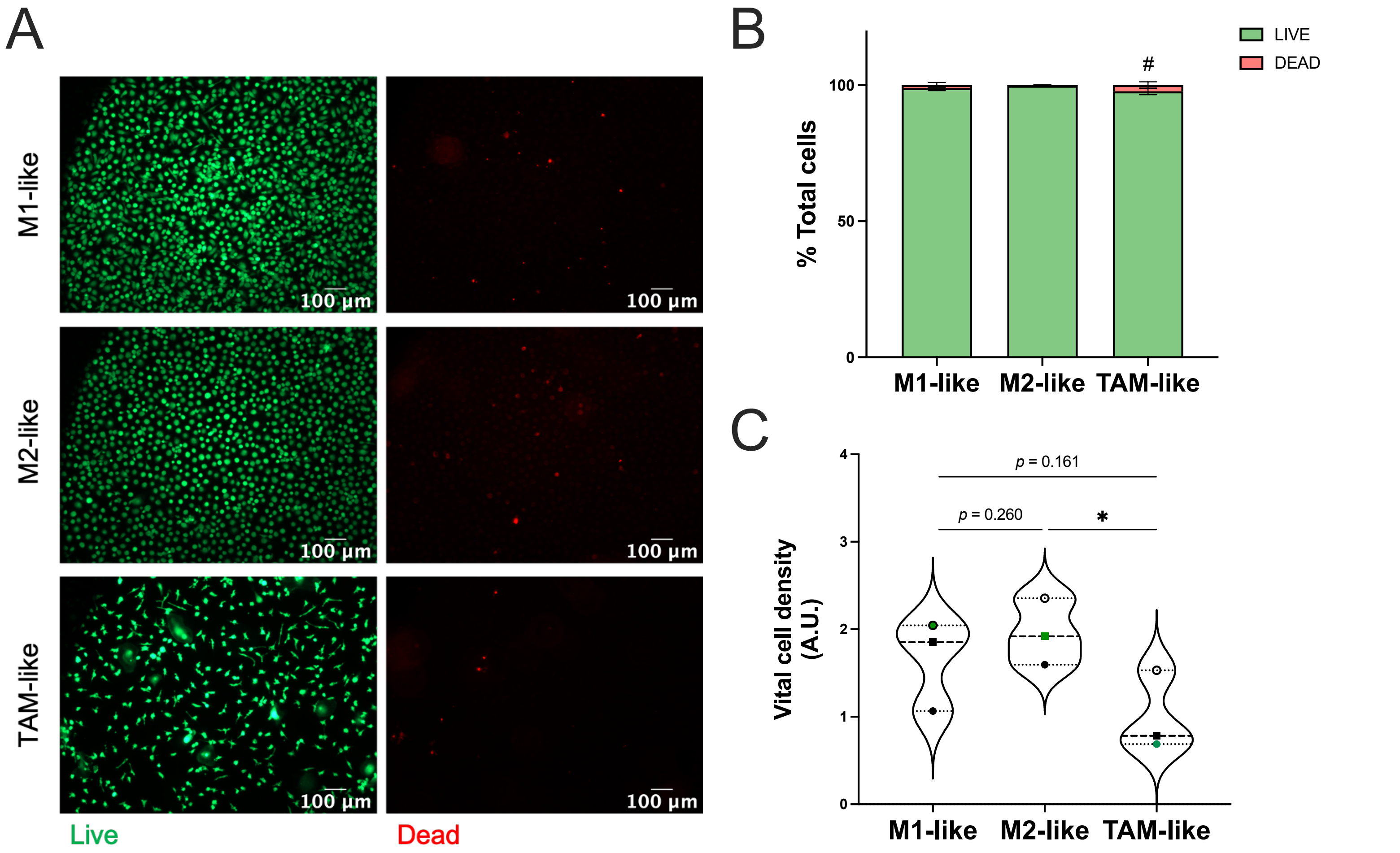


Figure S6
Pörschke *et al.*

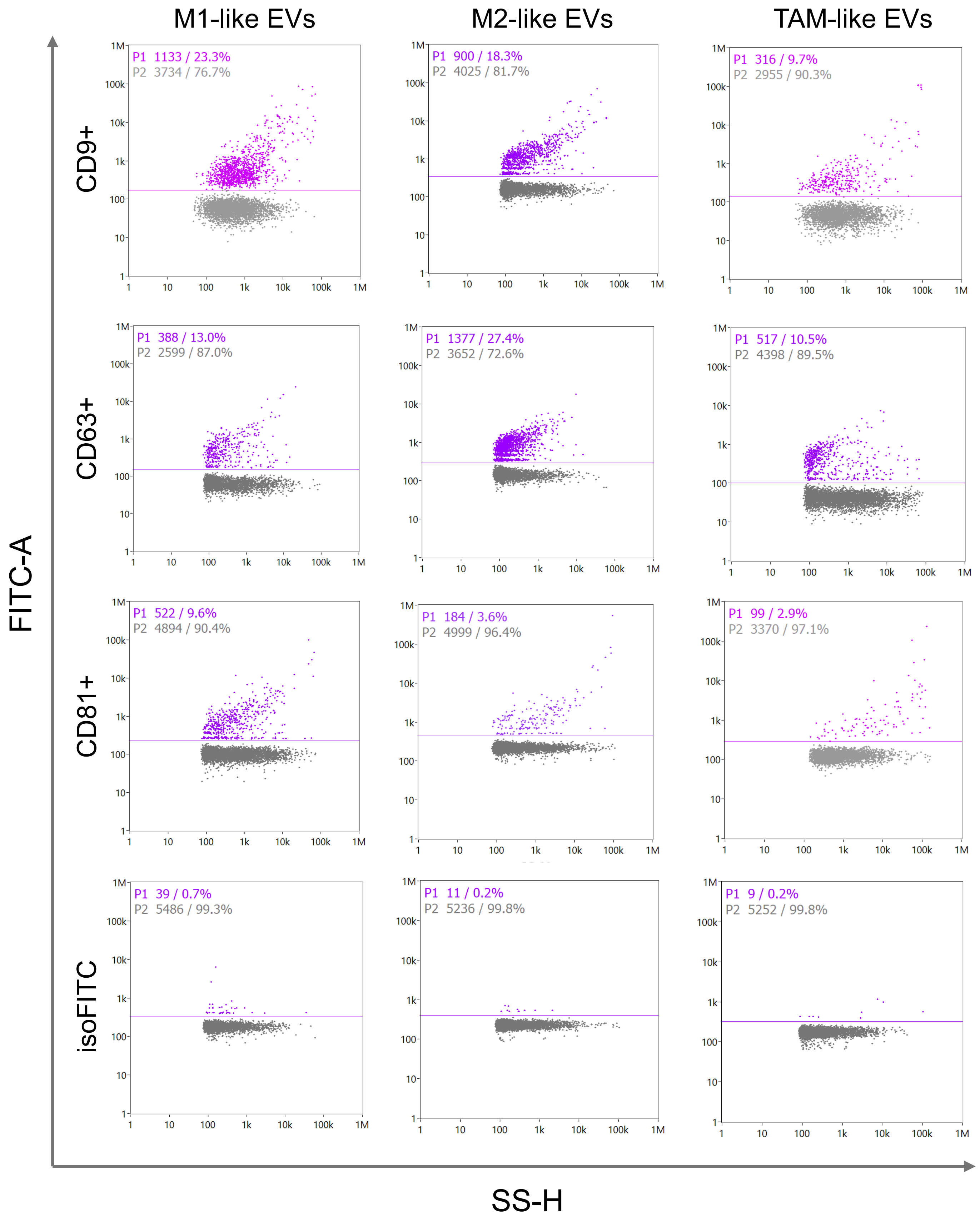


Figure S7
Pörschke *et al.*

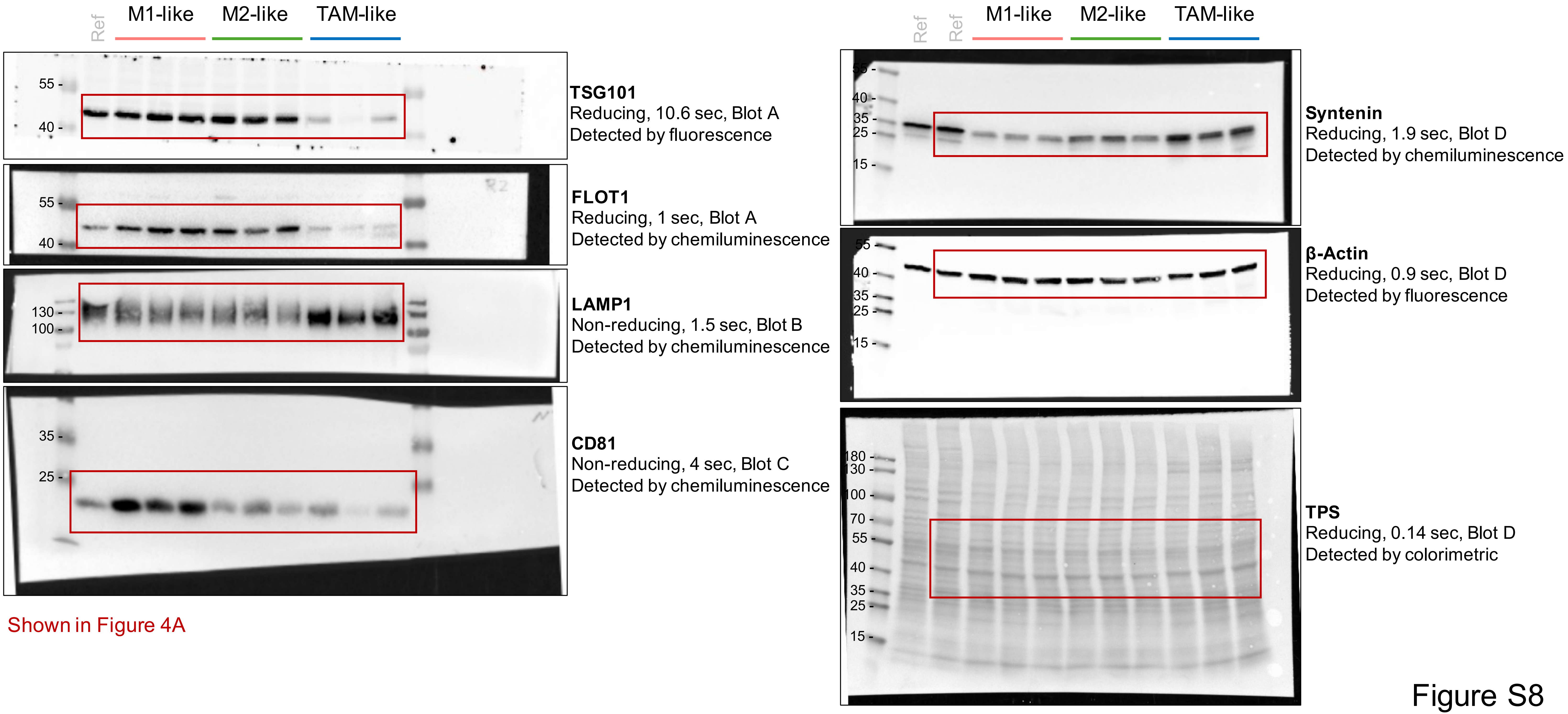


Figure S8
Pörschke *et al.*

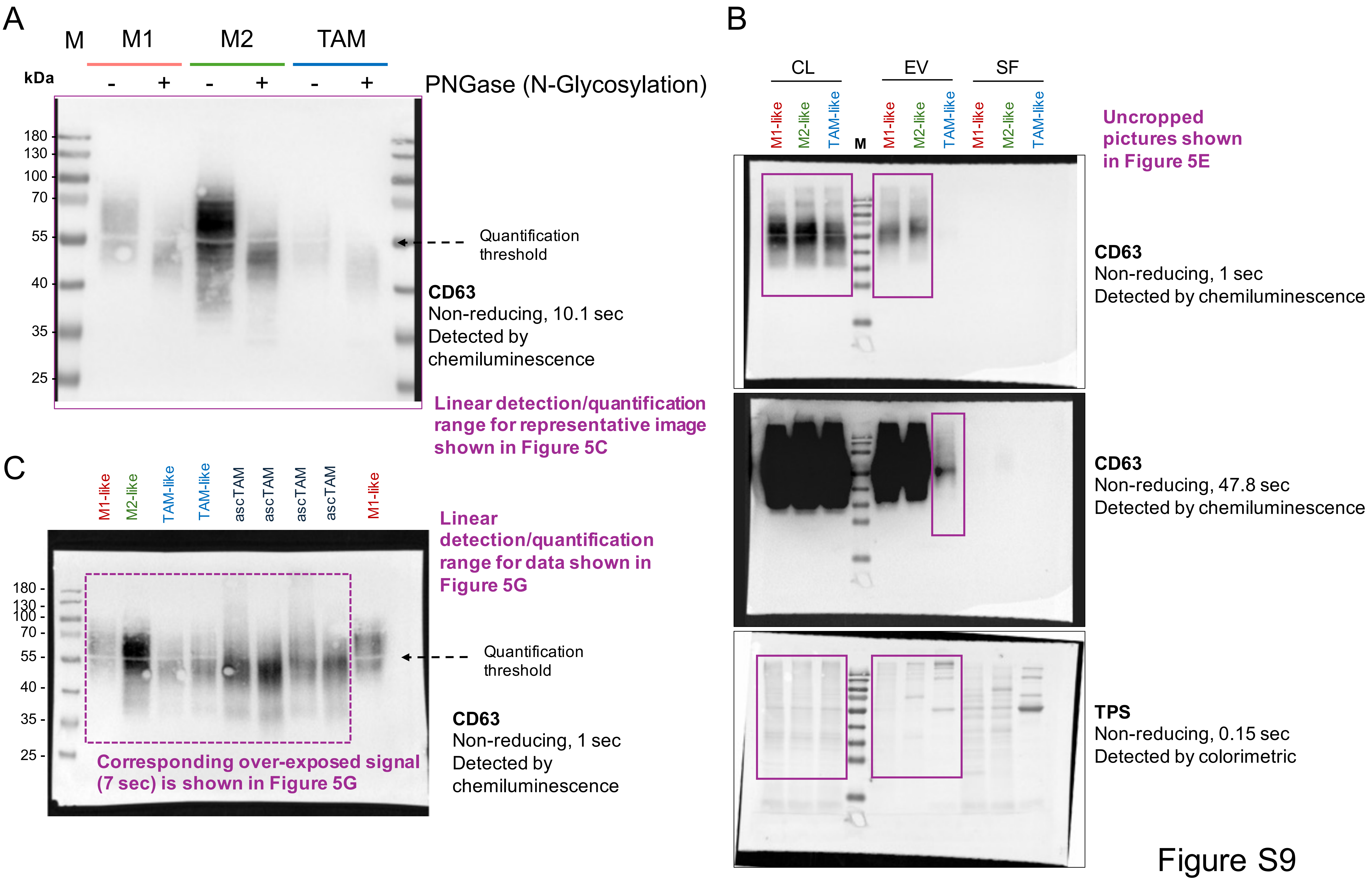


Figure S9
Pörschke *et al.*

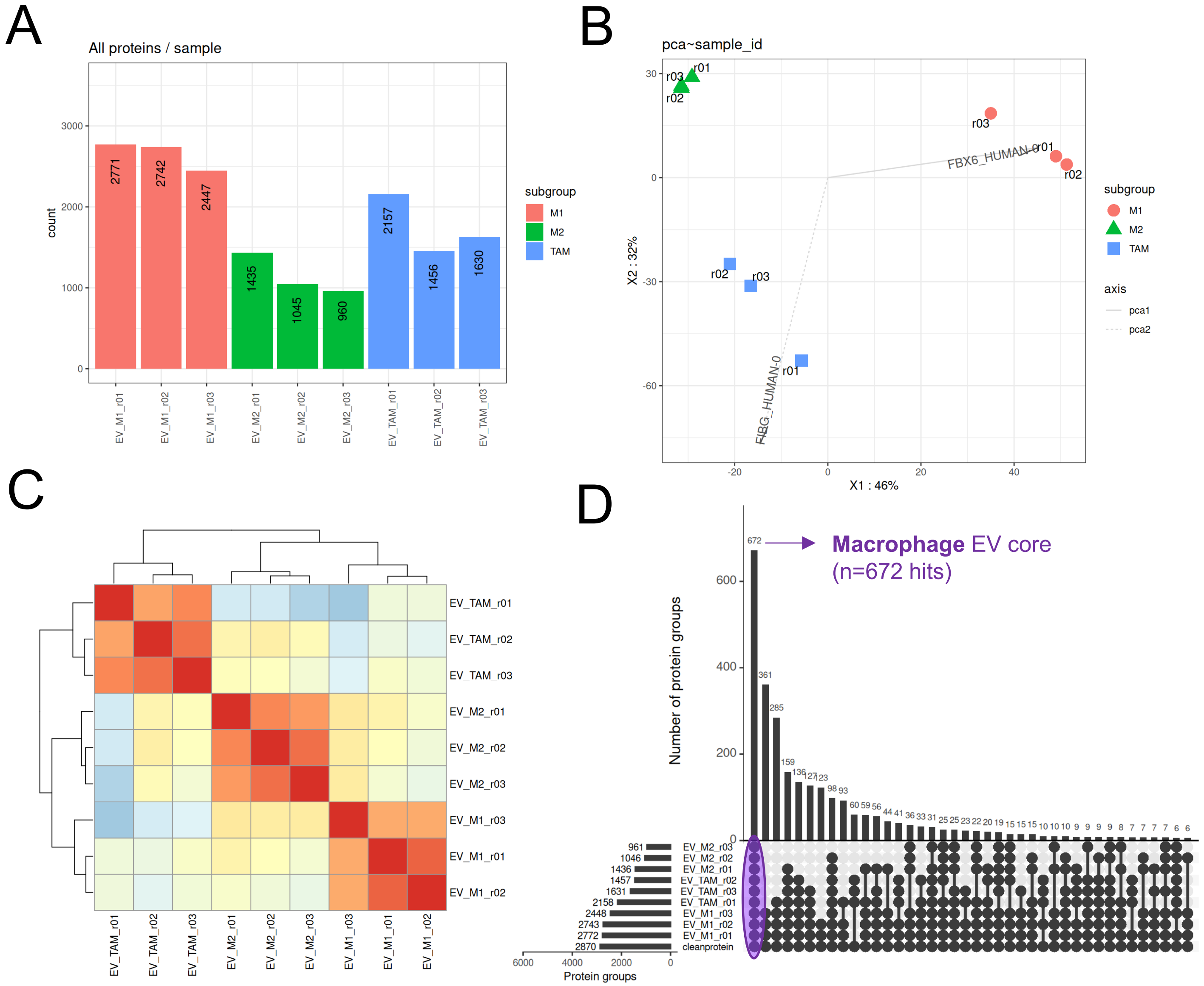


Figure S10
Pörschke *et al.*

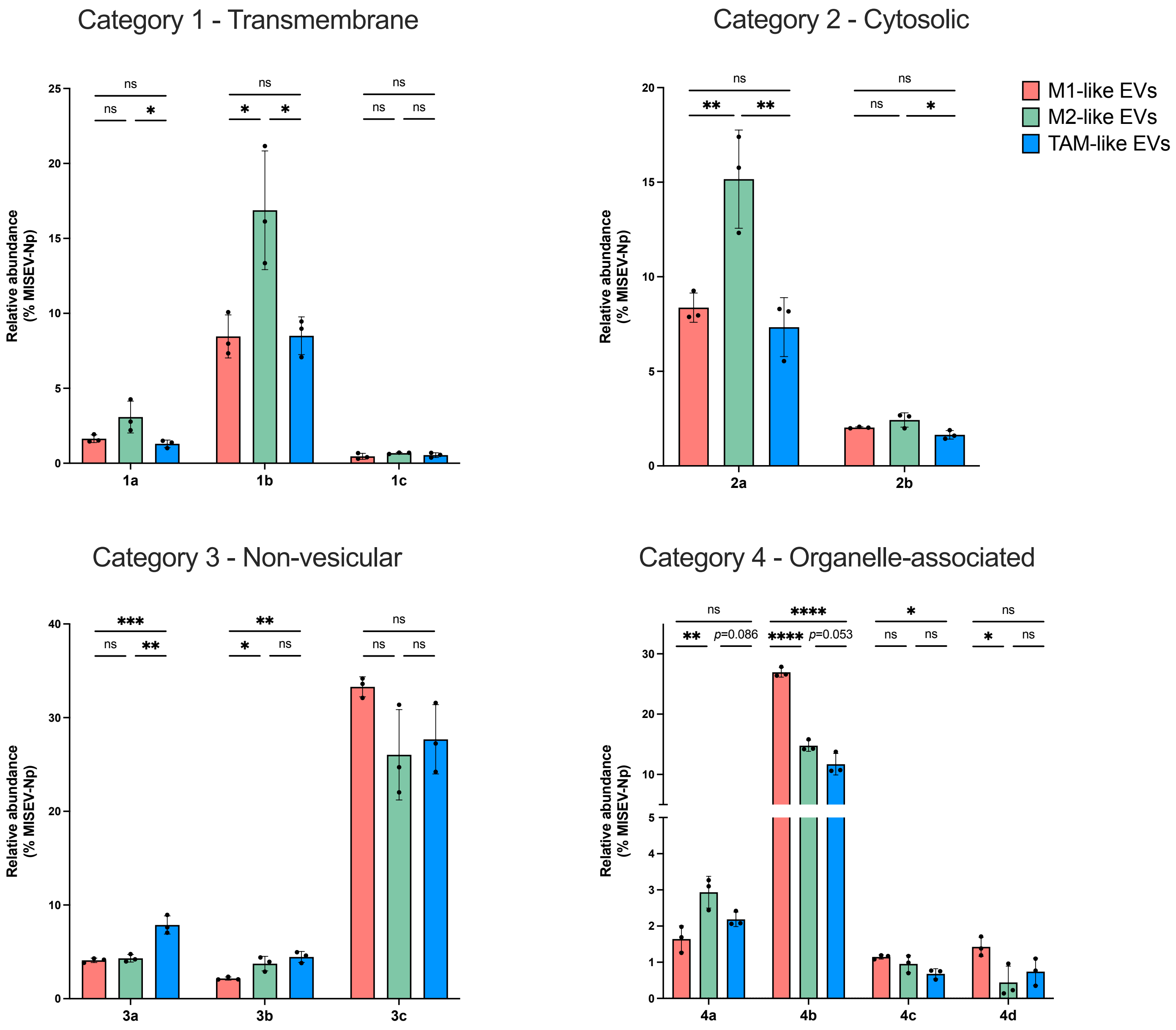


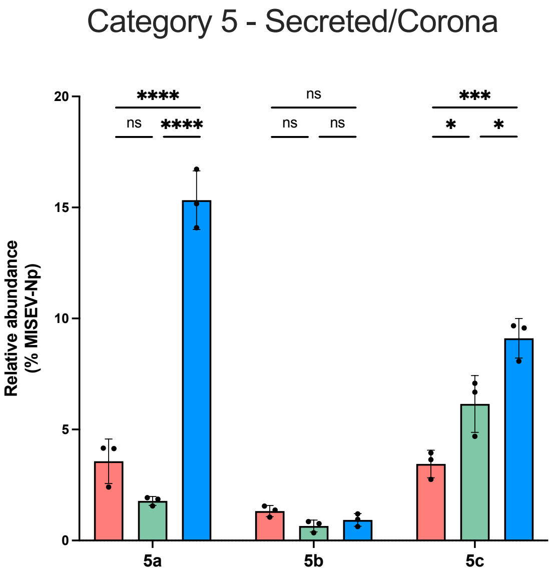


1a: multi-pass transmembrane proteins

1b: single-pass transmembrane proteins

1c: GPI- or lipid-anchored proteins

2a: with lipid or membrane protein-binding ability

2b: promiscous incorporation into EVs

3a: lipoproteins

3b: protein and protein/nucleic acid aggregates

3c: exomere or supermere-enriched components

4a: nuclear proteins

4b: mitochondrial proteins

4c: secretory pathway

4d: others

5a: blood-derived corona proteins

5b: cytokines and growth factors

5c: adhesion and extracellular matrix proteins

Figure S11
Pörschke *et al.*

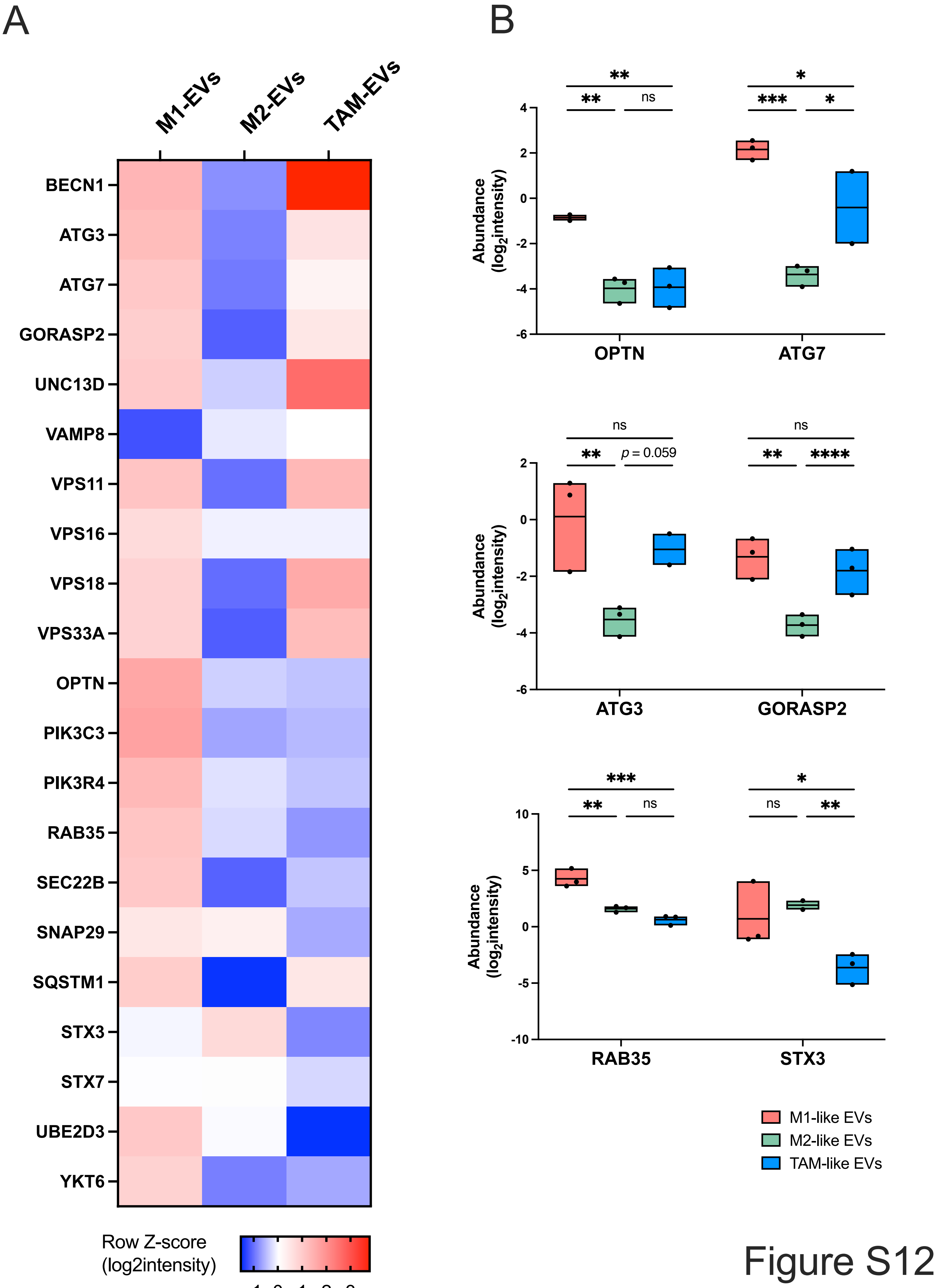


Figure S12
Pörschke *et al.*

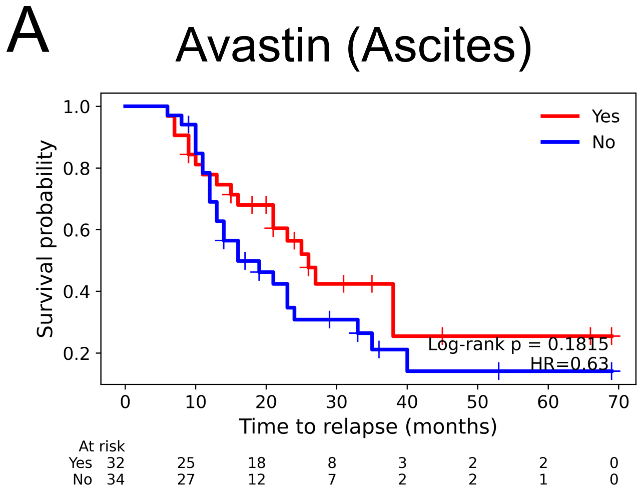


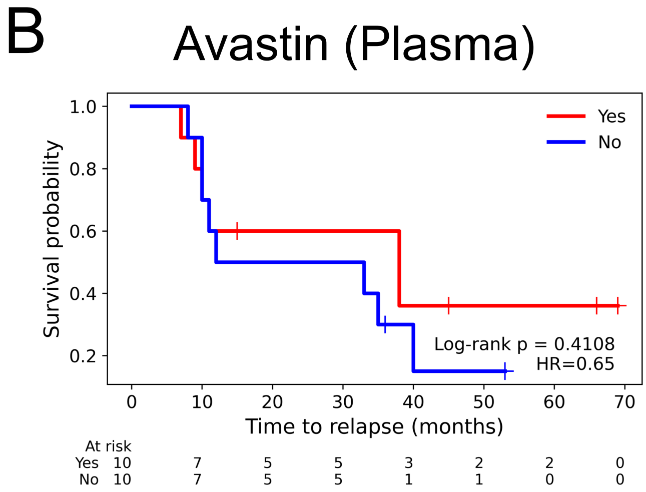


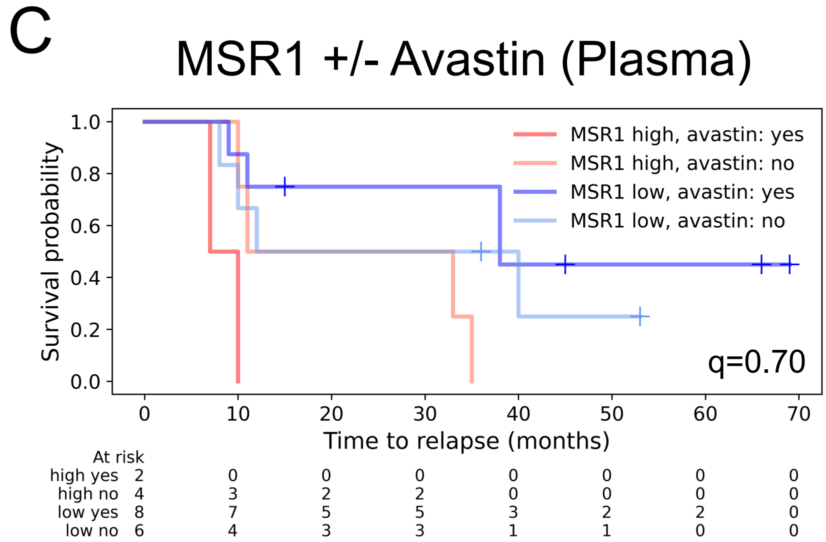


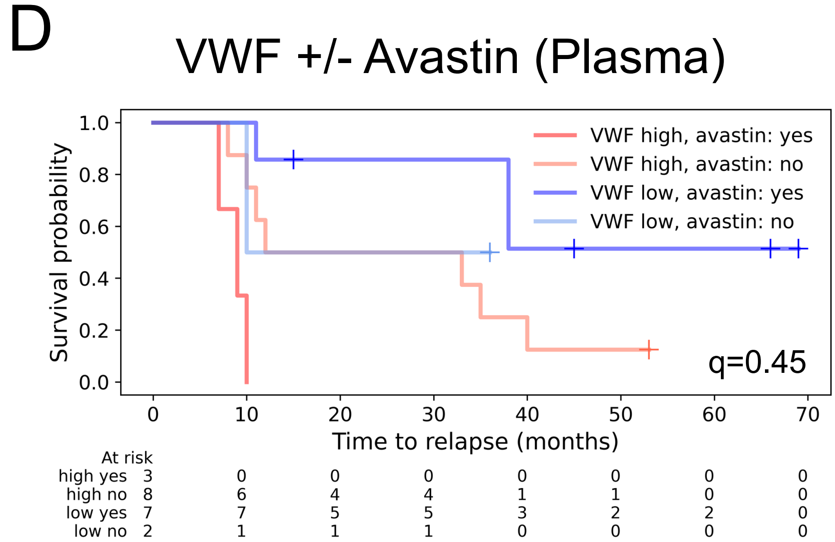


Figure S13
Pörschke *et al.*
