# Supplementary material for: Tumor-associated macrophages display differential protein cargo sorting in extracellular vesicles associated with poor survival in ovarian cancer
Source: Mol Med. 2026 Jan 30;32:16. doi: 10.1186/s10020-025-01416-x (PMC12875045; doi:10.1186/s10020-025-01416-x)
Supplement: Supplementary file 3 — Supplementary Material 3: Supplementary Information [file 10020_2025_1416_MOESM3_ESM.docx]

**Supplementary Information**

**Tumor-associated macrophages display differential protein cargo sorting in extracellular vesicles associated with poor survival in ovarian cancer**

Johanna Pörschke^1^, Sophie Heidemann^2^, Hannah P. Nehring^1§^, Aina Lluch^1^, Witold Szymański^3^, Florian Finkernagel^4^, Christian Preußer^1,5^, Aditya M. Bhagwat^3^, Timm J. Stamm^1$^, Leah Sommerfeld^2^, Frederik Helmprobst^6^, Rolf Müller^2^, Silke Reinartz^2^, Johannes Graumann^3^, Elke Pogge von Strandmann^1,5^, María Gómez-Serrano^1*^

^1^ Institute for Tumor Immunology, Center for Tumor Biology and Immunology (ZTI), Philipps-Universität, 35043 Marburg, Germany

^2^ Translational Oncology Group, Center for Tumor Biology and Immunology (ZTI), Philipps-Universität, 35043 Marburg, Germany

^3^ Institute of Translational Proteomics and Core Facility Translational Proteomics, Biochemical/Pharmacological Center, Philipps-Universität, 35043 Marburg, Germany

^4^ Genomics Core Facility, Philipps-Universität, 35043 Marburg, Germany

^5^ EV-iTEC Core Facility, Philipps-Universität, 35043 Marburg, Germany

^6^ Core Facility for Mouse Pathology and Electron Microscopy - Institute of Neuropathology, Philipps-Universität, 35043 Marburg

^§^ *Current address*: Department of Immunobiochemistry, Mannheim Institute for Innate Immunoscience (MI3) & European Center for Angioscience (ECAS), Medical Faculty Mannheim, Heidelberg University, Mannheim, Germany

^$^ *Current address*: Department of Urology, Universitätsklinikum Tübingen, 72076 Tübingen, Germany

* Corresponding author: [gomezser@staff.uni-marburg.de](mailto:gomezser@staff.uni-marburg.de)

**Supplementary Figures**

**Supplementary Figure S1. Effect of starvation of MDMs during 24h for EV collection.** Monocytes were isolated from peripheral blood of healthy blood donors and differentiated and polarized into macrophage subtypes (MDMs) upon cytokine or ascites treatment over seven days. Conditioned-media were collected after 24h using serum-free RPMI**. (A)** Morphology of the different MDM subtypes was recorded before (0h) and after starvation (24h). No remarkable differences could be observed over time. Representative images are shown. Magnification, 20X. Scale bar, 100 μm. **(B)** Flow-cytometry analysis of selected M1- (CD86, CCR7, HLA-DR) and M2-like (CD16, CD163, CD206) surface markers in the different macrophage subtypes after seven days of differentiation and subsequent incubation in RPMI serum-free media. Phenotypic changes before (t0, white bars) and after 24h starvation (grey bars) are depicted. The percentage of positive events is indicated, gated according to the isotype control. Mean + SD are shown, as well as individual values, corresponding to single donors (n=4). Statistical significance was tested by two-way ANOVA test (Bonferroni *post-hoc* test). For simplicity, only significant differences between t0 and t24h are indicated. D, donor; *, *p* value < 0.05; **, *p* value < 0.01; ***, *p* value < 0.001.

**Supplementary Figure S2.** **Uncropped pictures from WB results shown in Figure 2C.** Detection of different markers in MDM-derived EV preparations. EVs were isolated by differential centrifugation (UC). Non-EV (*i.e.,* calnexin, CANX), EV- (*i.e.,* Alix, FLOT1, Syntenin), and cytosolic markers (*i.e.,* GAPDH) were analyzed in M1-, M2- and TAM-like EV preparations by SDS-PAGE as described in the Material & Methods Section. Due to the low yield of MDM-derived EVs, around 1 μg of EV lysates could be analyzed. Comparative amounts of total cell lysates (CL) were analyzed (1 μg), together with higher levels of CL (5 μg) as a positive signal control. Total Protein Staining (TPS) was carried out to ensure equivalent loading. All markers were detected with different exposition times. FLOT1 and GAPDH were detected in the same membrane by chemiluminescence and fluorescence, respectively. M, marker.

**Supplementary Figure S3. Triton-X100 treatment of MDM-EV samples.** Data represent the fold-change of detected events by nano-flow-cytometry (nFC) of high-concentrated untreated (UT) and treated EV samples (n=5) isolated by ultracentrifugation (UC). Dilution within the linear range for sample measurement was done based on UT samples, and treated samples were correspondingly diluted. Triton-X100 solution was used as background control (< 100 events). Statistical significance was tested by one-sample *t*-test.

**Supplementary Figure S4. Particle release and size analyses of macrophage-derived conditioned media.** Conditioned media from different macrophage subtypes were collected after 24h starvation in addition to the corresponding time-point 0 h samples used as blank. **(A)** Particle concentration (No. particles/mL) was estimated by analyzing RPMI serum-free conditioned media (10,000 × g supernatants) by nano-flow cytometry (nFC). **(B)** Median size of analyzed particles (nm). The line indicates the mean values for all donors (n=6, indicated by different symbols). **(C)** Sizing profile of detected particles represented in panel B (bin size 10 nm). Non-linear gaussian fit curves are plotted for better visualization. Calculated means + SD are indicated. All measurements were done in technical duplicates (NanoFCM Software V1.08) and averaged values were tested for statistical significance by RM one-way ANOVA using Geisser-Greenhouse correction (Tukey *post-hoc* test). *, *p* value < 0.05; **, *p* value < 0.01.

**Supplementary Figure S5. Evaluation of particle release and size in UF-enriched EV samples from different macrophage subtypes.** Conditioned media (serum-free) from different macrophage subtypes were collected for 24h. Cell debris and large particles were removed and small particles were concentrated through ultra-filtration (UF) with AMICON devices (100 kDa cut-off). **(A)** Relative particle release was calculated by estimating the total number of particles collected per cell. Data are presented as fold-change relative to the mean of the TAM-like from all donors (n=5, indicated with different symbols). Differences between macrophage subtypes were assessed using the Friedman test followed by Dunn’s *post hoc* test. **(B)** Median size of analyzed particles (nm). The line indicates the mean values for all donors. All measurements were done in technical duplicates (NanoFCM Software V1.08). Statistical significance was tested by RM one-way ANOVA using Geisser-Greenhouse correction (followed by Tukey *post-hoc* test). *, *p* value < 0.05; **, *p* value < 0.01.

**Supplementary Figure S6. Evaluation of the vitality among different macrophages. (A)** Representative pictures of living (green) and dead (red) adherent macrophages after 24h starvation stained by *LIVE-DEAD Cell imaging kit* (Invitrogen). Magnification 10X. Scale bar 100 μm. **(B)** Relative proportion of living and dead cells. A total of 7 random fields per donor and MDM subtype were taken under Leica DMI3000 B Microscope and quantified by Image J. Averaged values were analysed and statistically tested by two-way ANOVA test (followed by Šídák's *post-hoc* test) (n=3 donors). #, *p* value < 0.05 for M2-like *vs*. TAM-like contrast. **(C)** Vital cell density detected in LIVE/DEAD cell staining pictures. Violin plots show the relative density of MDMs normalized to TAM-like average (n=3 donors). Mean differences were tested by by RM one-way ANOVA using Geisser-Greenhouse correction (followed by Tukey *post-hoc* test for multiple comparisons). *, *p* value < 0.05. Green symbols refer to donors showed in the representative pictures from panel A.

**Supplementary Figure S7. Gating strategy and representative nano-flow cytometry fluorescent results for the analysis of MDM-derived EVs.** UF-enriched samples (3-5 × 10^8^ particles) were stained for CD9, CD63 or CD81 using FITC-coupled antibodies. FITC-isotype control was used to exclude unspecific binding. Samples were washed with filtered PBS and ultracentrifuged prior to fluorescence detection. Representative scatter plots from EV-samples derived from the three macrophage subtypes are shown, depicting FITC area (FITC-A) on the y-axis and side scatter height (SS-H) on the x-axis (NanoFCM, V2.0). Positively stained events were gated in field P1 (purple) and unstained events are shown in P2 (grey). Percentages were used in bar plots shown in **Figure 3A** and sizing information were further processed with GraphPad Prism (V10.6.0) for histograms presented in **Figure 3B**.

**Supplementary Figure S8.** **Uncropped pictures from WB results shown in Figure 4A.** Detection of different markers in MDM cell lysates by SDS-PAGE and immunoblotting as described in the Material & Methods Section. Selected candidates were studied in MDM cellular lysates by SDS-PAGE combining reducing and non-reducing, denaturing conditions (for further details see **Supplementary Table S3**). Total Protein Staining (TPS) was carried out to ensure equal loading. Relative intensities were normalized to a reference sample (Ref) consisting on non-polarized, Mⱷ, MDMs. All markers were detected with different exposition times/methods as indicated. FLOT1 and TSG101, and Syntenin and β-Actin were detected in the same membrane by chemiluminescence and fluorescence, respectively.

**Supplementary Figure S9. Immunoblotting detection of CD63 protein in macrophages and EV lysates. (A)** Non-over-exposed image of CD63 detection in un-treated (-) or PNGase-treated (+) MDM whole cell protein lysates from a representative donor (corresponding to **Figure 5C**). Linear detection was used for relative quantification for each sample type (here M1- and M2-like cells, panel shown in **Figure 5C** for TAM-like cells). **(B)** Uncropped images shown in **Figure 5E**. Due to the differences in the relative abundance of CD63 in in macrophage-derived EV samples, different exposition times were combined. **(C)** Non-over-exposed image of CD63 detection in TAM lysates used for the quantification plotted in **Figure 5G**. CL, cell lysates; EV, EV-enriched samples obtained by ultrafiltration; M, marker; SF, soluble fraction obtained from UF processing; TPS, total protein staining.

**Supplementary Figure S10. Label-free mass spectrometry analyses of MDM-EV samples. (A)** Number of protein groups (protein IDs) identified in the individual replicates. A total of 2 × 10^9^ particles per replicate were used as input for protein digestion. Graph bars show the individual results for EV samples derived from M1-like (red), M2-like (green) and TAM-like (blue bars) macrophages. Full list is depicted in **Supplementary Table S5.** **(B)** Principal Component Analyses (PCA). Axes indicate the % of the total variance represented in the first (X1, 46%) and second (X2, 32%) components. r, replicate. **(C)** Protein correlation analyses. **(D)** Up-set plot showing the number of protein groups identified across replicates. In order to describe the “macrophage-EV core” (n=672 hits), only proteins IDs detected in all replicates were considered. Full list is depicted in **Supplementary Table S6.**

**Supplementary Figure S11. Relative abundance of MISEV annotated subcategories in the EV-associated proteome of macrophages.** Graph bars indicate the mean values (+/- SD) for EV samples derived from M1-like (red), M2-like (green) and TAM-like (blue bars) macrophages. Values represent the relative % of number of precursors (Np) of annotated proteins according to MISEV categorization. Statistical differences were tested by ordinary one-way ANOVA (Tukey´s *post-hoc* test). ns, non-significant, *p* value > 0.05; *, *p* value < 0.05; **, *p* value < 0.01; ***, *p* value < 0.001; ****, *p* value < 0.0001.

**Supplementary Figure S12. Abundance of autophagy-related proteins in MDM-EV samples. (A)** Heatmaps showing the relative abundance of autophagy-related proteins markers detected by label-free MS analyses. Color scale indicates the row Z-score (log_2_-normalized median intensity, n=3). **(B)** Box-plots indicating differential changes (log_2_-normalized intensity) of representative markers across macrophage-derived EV samples. Statistical significance was evaluated between groups by limma *t*-test (FDR-adjusted *p* value). *, *p* < 0.05; **, *p* < 0.01; ***, *p* < 0.001; ****, *p* < 0.0001. ATG7, Autophagy-related protein 7; GORASP2, Golgi reassembly-stacking protein 2); OPTN, optineurin; RAB35, Ras-related protein Rab-35; STX3, Syntaxin 3.

**Supplementary Figure S13. Evaluation of potential biases derived from Avastin® (bevacizumab) treatment in RFS associations.** Kaplan-Meier plots show the association of Avastin® treatment with the survival of HGSC patients whose **(A)** ascites (n=70) or **(B)** plasma (n=20) samples were previously analyzed by affinity proteomics (62,63). Association of MSR1 **(C)** and VWF **(D)** levels in plasma in patients treated or not with Avastin®. Quantiles used for splitting datasets (high/low) were the same applied in **Figure 8E**. Due to the low number of patients per group, statistical evaluation in panels C, D was omitted. Red and blue lines indicate the use of Avastin® as maintenance therapy or not, respectively. HR, hazard-ratio.

**Supplementary Tables (Spreadsheets)**

| **Table S1** | Patient characteristics. Maintenance therapy based on Avastin® (bevacizumab) or poly (ADP-ribose) polymerase inhibitors (PARPi) is indicated. N/A, not applicable. |
| --- | --- |
| **Table S2** | Ascites pools used in this study. Cell-free ascites fluid was used to induce the polarization of monocyte-derived macrophages (MDMs) towards a tumor-associated-macrophage (TAM) phenotype (ascMDMs / TAM-like cells). Ascites pools were created by pooling equal volumes of fluid (n=10 patients) that were subsequently aliquoted and stored at -80°C until use for TAM-like polarization experiments. Between batches, patient-composition slightly fluctuates, while characteristics of the patients are balanced. **(A)** Pools of cell-free ascites fluid used in this work. Orthogonal methods are indicated. **(B)** Patient characteristics of three representative ascites pools. EM, electron microscopy; nFC, nano-flow cytometry; WB, western-blot. |
| **Table S3** | List of primary antibodies and conditions used for immunoblotting. |
| **Table S4** | Reference MISEV annotation list for human genes/proteins into main and subcategories. |
| **Table S5** | Proteins detected in EV samples from M1-, M2- and TAM-like EVs. The Table shows the log2 normalized intensity values for the protein groups (n=2,870) detected among the different samples and replicates (3 replicates: R1-R3). Corresponding plot is shown in **Supplementary Figure S10A**. |
| **Table S6** | The macrophage-EV core proteome. This subset was defined for proteins detected in EVs from all macrophage subtypes and replicates (n=672 hits). Corresponding plot is shown in **Supplementary Figure S10D**. |
| **Table S7** | MISEV annotation categories and corresponding match in our dataset. The table indicates the number of protein groups (protein IDs) detected for each (sub-)category for MDM-EV samples. Filled cells indicate those subcategories considered for endosomal-related release estimation. |
| **Table S8** | Pro- and anti-inflammatory protein component of macrophage-derived EVs. Differentially abundant proteins (DAPs) between M1- and M2-like derived EVs. Abundance ratio is expressed as log2 fold-change of the normalized intensity values. Only protein groups showing significant changes (FDR-adjusted *p* value) are shown. TRUE/FALSE indicate imputed and non-imputed values for statistical evaluation. Corresponding volcano plot is shown in **Figure 7A**. |
| **Table S9** | TAM-enriched protein component of EVs. Differentially abundant proteins (DAPs) between TAM and M1- or M2-like derived EVs. Abundance ratio is expressed as log2 fold-change of the normalized intensity values between the conditions. Only protein groups showing significant changes (FDR-adjusted *p* value) are shown. TRUE/FALSE indicate imputed and non-imputed values for statistical evaluation. Note that this list contains n=197 hits which were DAPs in both differential studies (indicated as "overlapping"). Corresponding volcano plots are shown in **Figure 7B**. |
| **Table S10** | Macrophage signatures of the EV-associated proteome. Lists of differentially abundant proteins (DAPs) depicted in **Figure 7C**. Associated, corresponding data in previously published datasets are also shown. Terms belonging to the "TAM-EV signature (n=225) were used for over-representation analyses (ORA) shown in **Figure 7D**. |
| **Table S11** | Over-representation analyses (ORA) of *Gene Ontology* (GO) terms for the TAM-EV signature panel (n=225 proteins). Only significantly enriched terms (adjusted *p* value < 0.05) included in the ontology classification for *Biological Process* (BP), *Cellular Component* (CC) or *Molecular Function* (MF) are reported below. Corresponding terms are shown in **Figure 7D**. Highlighted terms (bold) indicate those processes including TAM-EV proteins with translational relevance at circulating levels (see **Figure 8B**, significantly abundant in OC *vs*. non-cancer patients). |
